# Determinants of adoption of climate-smart agriculture (CSA) practices in mushroom farming in Bangladesh

**DOI:** 10.1038/s41598-026-39761-4

**Published:** 2026-02-19

**Authors:** Iqramul Haq, Mofasser Rahman, Tanmay Datta, Mahmudul Hassan Rakib, Diego Nobrega

**Affiliations:** 1 Faculty of Veterinary Medicine, University of Calgary, Calgary, AB T2N 1N4 Canada; 2 Department of Agricultural Statistics, Sher-e-Bangla Agricultural University, Dhaka, 1207 Bangladesh; 3 Department of Agribusiness and Marketing, Sher-e-Bangla Agricultural University, Dhaka, 1207 Bangladesh; 4 Department of Agricultural Economics, Sher-e-Bangla Agricultural University, Dhaka, 1207 Bangladesh

**Keywords:** Agriculture, Climate change, Climate-smart agriculture, Mushroom farming, Precision technology, Climate sciences, Ecology, Ecology, Environmental sciences, Environmental social sciences, Plant sciences

## Abstract

Climate-Smart Agriculture (CSA) practices can reduce effects of climate change in agriculture by increasing production efficiency. Yet, adoption of CSA practices is not universal on Bangladesh farms. This study aim is to identify determinants of adoption of CSA practices in mushroom farming in Bangladesh using a combination of frequentist approaches and machine learning (ML). A total 150 mushroom farmers were selected from Savar upazila. Farmers were interviewed using a questionnaire, and results were analyzed using a combination of Bayesian and ML approaches. Among respondents, 48% of farmers had adopted at least one CSA practice for mushroom farming. Bayesian analysis revealed that mushroom farmers with secondary education, prior knowledge of CSA, training related to mushroom cultivation, access to climate information and credit were more likely to adopt CSA practice for mushroom production compared to their peers. Additionally, new farmers had higher odds of adopting CSA than their counterparts. In terms of predicting adoption of CSA practices using ML, the support vector machine algorithm slightly outperformed other ML algorithms, with an estimated accuracy of 87.3%, recall of 92%, F-score of 87.9%, g-mean score of 87.7%, Cohen’s Kappa of 74.6%, and Matthews’ correlation coefficient of 74.7%. However, for area under the curve and precision recall curve, gradient boosting machine showed better performance. Consistent with the frequentist approach, prior knowledge of CSA practices was among the most influent factors towards adoption of CSA practices for mushroom farming, followed by access to climate information, farm ownership, training related to mushroom, access to credit and internet. We hypothesize that targeted training, and access to climate information and credit will increase adoption of CSA practices in mushroom farming in Bangladesh.

**Supplementary Information:**

The online version contains supplementary material available at 10.1038/s41598-026-39761-4.

## Introduction

Mushrooms are a relevant source of nutrients, tonics, and medicines^1^. As mushrooms are grown in vast quantities quickly and offer the highest protein output per unit area among crops, they can help reduce malnutrition^2^. Due to climate, reduced production costs, availability of growing substrates, and a strong market demand, Bangladesh offers a suitable environment for mushroom cultivation^3,4^. In Bangladesh, mushroom farming as a business is a growing concept among young, educated people and rural women^2^. Small family businesses that lack sufficient acreage to cultivate crops and rear animals find intensive mushroom growing to be an excellent source of alternative revenue^5^.

Climate change is a global threat^6^. The average temperature in Bangladesh has been trending upward, with the rainy season expected to become wetter and the dry season becoming drier^7^. Additionally, extreme climate events are more likely in face of climate change. Climate-smart agriculture (CSA) promotes sustainable farming by enhancing productivity, resilience, and environmental sustainability to support global food security^8^. In this context, CSA ideas and methods are becoming increasingly popular worldwide to meet the challenges in agricultural productivity in face of climate change^9,10^. Mushroom production significantly contributes to greenhouse gas emissions, mainly from non-renewable energy and transportation^11^. CSA adoption is associated with lower greenhouse gas emissions and water pollution while increasing rice grain output, decreasing urea prices, and reducing nitrogen loss by 40%^12^, aligning with the UN sustainable development goals^13^. To date, research exploring determinants of CSA practice in mushroom production specifically is scarce.

Traditional statistical approaches have also been applied to analyze factors affecting mushroom production and profitability. In Bangladesh, multinomial logistic regression was applied to identify key determinants of mushroom cultivation^14^, while multiple linear regression examined factors affecting mushroom farm profitability^15^. Additionally, climate-related studies have explored the relationship between environmental variables and mushroom production. Generalized additive models have been employed to analyze how climate factors affect mushroom production over time^16^. In Spain, linear regression and correlation analysis examined the relationship between mushroom yields and climatic variables^17^, while mixed-effects models were utilized to predict annual mushroom occurrence and yield based on weather conditions such as precipitation and soil moisture^18^. Economic modeling techniques have been used to understand management decisions and consumption behavior in mushroom farming. In Ghana, an ordered probit model was applied to analyze the determinants of mushroom consumption^19^. Similarly, in Swaziland, two-stage probit least squares and conditional maximum likelihood estimation were used to identify factors influencing farmers’ decisions to produce oyster mushrooms^20^. Incorporating technologies like artificial intelligence, machine learning (ML), and deep learning (DL) can reduce energy use and improve production and logistics in mushroom cultivation^21,22^. Artificial Neural Networks, Adaptive Neuro-Fuzzy Inference Systems, and Naïve Bayes (NB) classifiers were used to distinguish between edible and non-edible mushrooms^23,24^. Convolutional neural network (CNN) algorithms have been employed to predict and classify oyster mushroom diseases^25^. A systematic review provided insights into ML trends, evaluation techniques, data sources, and methodologies in mushroom farming^26^.

This study aim is to identify key factors influencing CSA practice adoption among mushroom farmers in Savar Upazila, Bangladesh, using a combination of frequentist and ML approaches, with the goal of identifying elements that can stimulate the increased adoption of CSA practice in mushroom farming in Bangladesh.

## Methodology

### Sources of data and study period

**Fig. 1:**
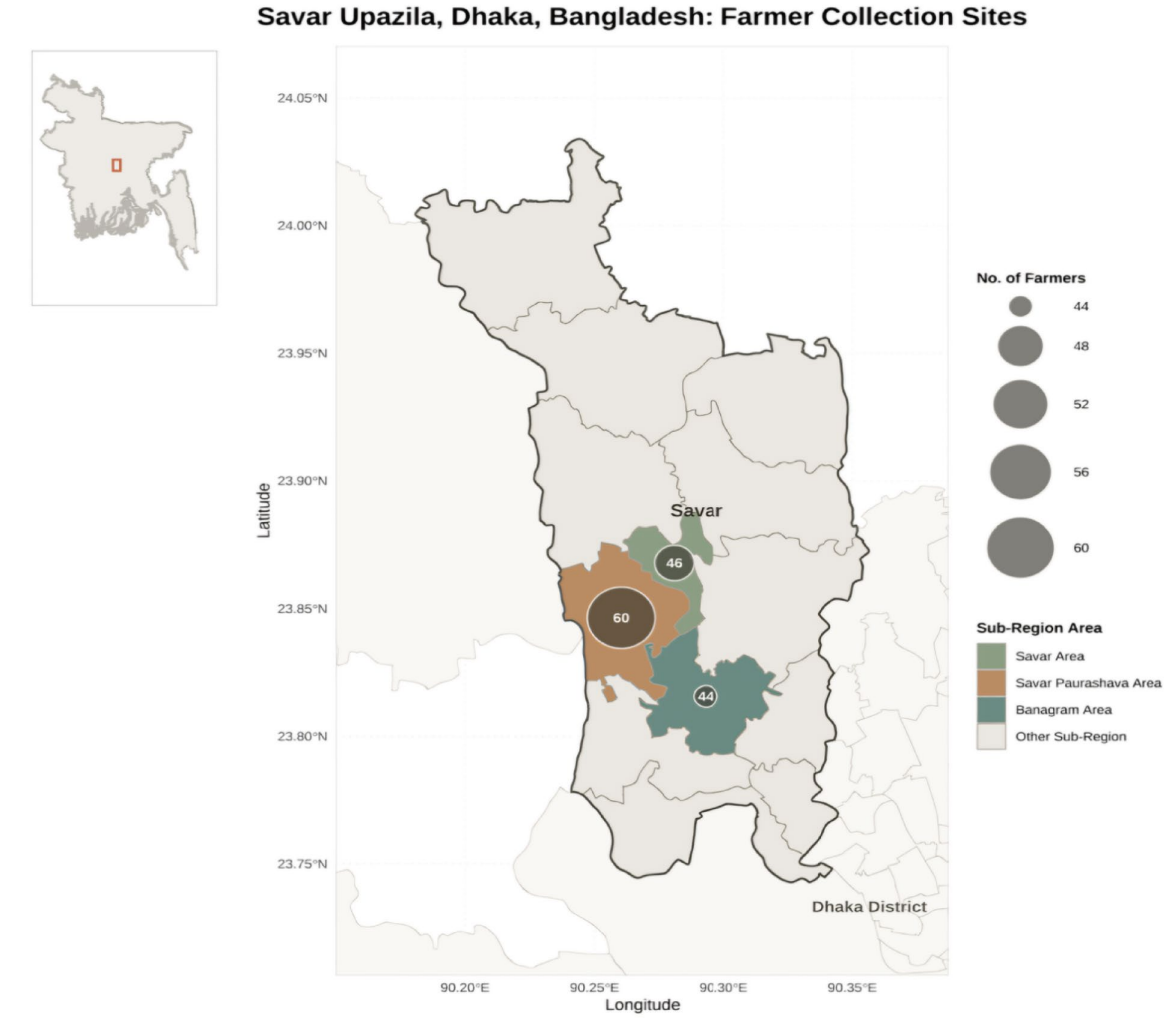
Geographic map of Savar Upazila, Dhaka, Bangladesh, showing data collection sites across sub−regions. Map created by the authors using R software (version 4.4.0; https://www.r-project.org/) and the packages *sf, ggplot2, cowplot, viridis*, and *dplyr*.

This cross-sectional study was conducted during 2023–2024, focusing on collecting data from mushroom farmers in the primary mushroom-producing upazilas within the Dhaka district. Since Savar Upazila accounts for a significant proportion of mushroom production in Bangladesh, the study was limited to farmers located in this area (Fig. 1).

### Study type and study population

A cross-sectional survey of mushroom farmers was conducted. The study population consisted of farmers who were directly engaged in mushroom cultivation in the Dhaka district.

### Sampling design

The survey used a multistage stratified sampling technique. At the initial stage, Savar Upazila was purposively selected due to the large number of households in the area that rely on mushroom production as their primary source of livelihood.

In the second stage, using a list of farmers obtained from Upazila Agricultural Office in Savar, three major mushroom-producing areas were randomly chosen from Savar Upazila. In the third stage, a random number generator was used to select 60 mushroom farmers from Savar Paurashava area, 46 from Savar area, and 44 from Banagram area.

### Sample size

We calculated the minimum required number of completed interviews using the following formula:

$$\:n=\frac{{Z}^{2}pq}{{d}^{2}}\times\:deff$$

Where, n = required sample size.

z = 1.96, corresponding to a 95% confidence level.

p = estimated proportion in the survey = 0.50.

q= (1-p) = 0.50.

d = desired level of precision in the estimate = 0.05.

and deff.= design effect for multistage stratified sampling set at 0.4.

resulting in $\:\approx\:$150. Assuming a 10% non-response rate, we therefore targeted 165 farmers; 150 completed interviews were included in the final analysis. 

### Ethical statement

The Department of Agricultural Statistics, Sher-e-Bangla Agricultural University (SAU), Dhaka-1207, Bangladesh, reviewed and approved this research, confirming its compliance with the ethical guidelines stipulated by the university. The ethical approval was granted under the reference number SAU/AGST/2023/243(1). The goal of this study was explained to all farmers prior to data collection. Farmers’ anonymity and data confidentiality were strictly maintained, and informed consent was obtained from all respondents. All methods were carried out in accordance with the relevant guidelines and regulations, and in compliance with the principles of the Declaration of Helsinki (1975, revised 2013).

### Methods of data collection

A paper-based, structured questionnaire was administered to gather relevant information from mushroom farmers. Instructors provided clear explanations of the study’s purpose before asking farmers to complete the questionnaire. The questions were presented in both English and Bengali, languages that respondents were fluent in. Data collection was carried out through face-to-face interviews using the paper-based questionnaire (see the supplementary questionnaires: English, pages 17–19; Bengali, pages 20–23). Before initiating data collection, the questionnaire was reviewed by an expert in CSA (MMRS, Chairman, Department of Agricultural Statistics, SAU) for further refinement.

### Pretest the questionnaire

Pre-testing the questionnaire is a critical step in survey development. It allows investigators to evaluate how well respondents understood questions and whether they could provide the required information or complete the tasks as intended. Our initial test focused on clarity of the language, time required for administration, and respondents’ comprehension of general statements. This pre-test was conducted with a group of 10 farmers. Based on their feedback, no major modifications were implemented in the questionnaire, and the document was deemed clear and suitable for the main survey.

### Response variable

Based on the literature, we identified key CSA practices for mushroom cultivation (^27–32^; Javier Alejandro^33–39^). The binary dependent variable in this study was farmers’ adoption of any CSA practice in mushroom farming. This variable was categorized into two groups: those who adopted at least one CSA practice and those who did not. CSA practices included the following: utilization of an autoclave for sterilizing mushroom substrates, adoption of climate-friendly varieties (oyster mushrooms, and king oyster), use of high yielding mushroom varieties (*Pleurotus* spp*.* and *Pleurotus eryngii*), utilization of Internet of Things (IoT)-based monitoring and control systems, use of organic fertilizer, climate control via heating, ventilation, and air conditioning (HVAC) equipment, adoption of solar energy (e.g., photovoltaic (PV) for mushroom cultivation, use of electric sterilizers (steam) machines for bag filling, humidity control via spraying water mist using spray, utilization of integrated pest management (IPM) practices, use of sprinklers for optimal humidity level, use of media (sawdust and rice straw) as compost for mushroom production, and use of spent mushroom substrate (SMS) as an alternative method for burying media and polythene covers. Non-CSA group included respondents who did not use any CSA practice on their farms.

$$\text{CSA practice}\;=\;\begin{cases}\text{Yes, if the farmer adopts at least one CSA practice for mushroom farming}\\\text{No, otherwise}\end{cases}$$

For descriptive purposes, each listed practice was systematically classified according to the three core CSA pillars based on the FAO definition^40^ practices that aim to sustainably increase productivity, strengthen resilience (adaptation), or reduce or remove greenhouse gas emissions (mitigation), as supported by literature. Practices such as IoT-based monitoring systems, organic fertilizer application, use of electric steam sterilizers for bag filling, use of media (sawdust and rice straw) as compost for mushroom production, and IPM contribute primarily to adaptation by enhancing resilience and resource efficiency^36,41–45^. Technologies including solar energy systems, IoT-based monitoring, electric sterilizers, climate-controlled (HVAC) facilities, and use of SMS as an alternative method for burying media and polythene covers were classified under mitigation, as they reduce reliance on non-renewable inputs and lower greenhouse gas emissions^28,32,46–49^. Finally, practices such as the adoption of high-yielding and climate-friendly mushroom varieties, use of autoclave sterilizing for substrates, humidity control via water mist spray, and the use of sprinklers for optimal humidity levels were classified under productivity, as they directly enhance yield and efficiency^29,36,50–54^. The overall classification of all practices under each pillar is presented in Supplemental Fig. 1. The adoption of CSA pillars among mushroom farmers is summarized in Supplemental Table 1. The Adaptation pillar recorded the highest adoption (42.6%), followed by productivity (41.2%), while Mitigation was least adopted (16.2%). At the practice level, the most common measures included high-yielding varieties (44.7%), organic fertilizer use (43.3%), and compost media from sawdust and rice straw (38.7%). In contrast, energy-intensive practices such as IoT-based monitoring systems (4.0%), HVAC control (5.3%), and electric steam sterilizers (5.3%) were less common, indicating that farmers favored low-cost, resilience- and productivity-enhancing practices over capital-intensive mitigation technologies.

### Independent variables

In addition to the dependent variable, a range of socio-demographic factors were considered as covariates. These factors included the farmer’s age (< 40 and 40+), education level (no education, primary and secondary+), gender (male and female), family size, area (Savar, Savar Paurashava and Banagram), mushroom farming experience (years) (< 5 and 5+), mushroom farming in own land (no and yes), prior knowledge about CSA practices (no and yes), internet access (no and yes), access to market (no and yes), knowledge about relative humidity and ventilation for mushroom cultivation (no and yes), access to climatic information for mushroom cultivation (no and yes), regular extension visits (no and yes), contract farming (no and yes), transport availability (no and yes), prior training for mushroom farming (no and yes), access to credit (no and yes), membership of farming group (no and yes), mobile phone ownership (no and yes), off farm job opportunity (no and yes) and local storage facilities for mushroom farming(no and yes). Additionally, answers to the following questions were also investigated as independent variables:

- Do you know the appropriate temperature required for mushroom farming? (no and yes);
- Do you know the importance of substrate (soil) quality in mushroom cultivation? (no and yes);
- Do you have access to climatic information (e.g., temperature, humidity) for mushroom cultivation? (no and yes);
- Do you know the importance of light in mushroom cultivation? (no and yes).

### Statistical analysis

Data were thoroughly checked and verified to minimize inconsistencies. Descriptive analysis involved calculating frequency distributions and percentages. To initially assess the association between the response variable and covariates, a chi-square test was applied. Thereafter, we applied the Boruta algorithm for feature selection, and Cramér’s V correlation to identify the associations among the selected covariates. The Boruta algorithm (available in the *Boruta* package in R) is a feature selection method that uses random forest (RF) to identify all relevant variables in a dataset. Next, two approaches were used to investigate associations between selected features and CSA adoption: Bayesian logistic regression analysis and ML.

### Bayesian logistic regression

In the Bayesian model, posterior estimation was performed using the Hamiltonian Monte Carlo (HMC) algorithm within the Markov Chain Monte Carlo (MCMC) framework^55–57^. The PyMC^58^ and Bambi^59^and ArviZ^60^Python packages were used^59^. If prior knowledge was lacking, we assigned weak prior to regression coefficients- $\:{\beta\:}_{j\:}\sim\:N\:(0,{\:0.5}^{2})$ where $\:{\beta\:}_{j\:}$is the regression coefficient for predictor *j* with mean 0 and standard deviation 0.5. Similarly, the intercept followed a normal informative prior with mean 0 and standard deviation 0.5. The posterior distributions were consistently narrower and more peaked than priors, indicating that the data substantially reduced uncertainty in parameter estimates (supplemental Fig. 2).

To ensure robust posterior estimation, we utilized an HMC chain with 100,000 iterations per chain. We used four chain and produce total 400,000 posterior samples while for adapting the No-U-Turn Sampler (NUTS) sampler. A random seed (1234) was set.

We used the Gelman-Rubin convergence criterion ($\:\widehat{R}$) to determine whether Markov chains have reached stationary distributions. An $\:\widehat{R}$ value of 1 indicates convergence^61^. We also monitored effective sample size (n_eff), the R-hat statistic, and trace plots to evaluate model efficiency, convergence and fit^62–64^.

For model fitness we used widely applicable information criterion (WAIC) and leave-one-out cross-validation (LOO-CV). The highest density interval (HDI) is a widely used measure of uncertainty in Bayesian inference^65,66^. We estimated adjusted odds ratios (aOR) with 95% HDI from the final model. Posterior distribution of parameter estimates are shows in supplemental Fig. 3. Data analysis was conducted using R 4.4.0 (https://www.r-project.org/) and Python package version 3.13.5 (https://www.python.org/).

### Machine learning

We used nine of the most popular ML algorithms (logistic regression (LR), RF, support vector machine (SVM), decision Tree (DT), gradient boosting machine (GBM), Histogram-based Gradient Boosting Machine (HistGBM), DL-Multilayer Perceptron (DL-MLP), eXtreme Gradient Boosting (XGBoost) and Adaptive Boosting (AdaBoost) to explore determinants of adoption of CSA practices in mushroom cultivation in Savar Upazila.

### Data preparation and hyperparameter

Since our sample size was small, we used repeated stratified 10-fold cross-validation (CV) for the ML approach.​ Data preprocessing was carried out using Python, Scikit-learn, Pandas, and Keras^67–69^. To address class imbalance in the dataset, we applied the Synthetic Minority Over-sampling Technique (SMOTE). SMOTE improves classification on imbalanced data by generating synthetic minority samples through interpolation between existing samples and their nearest neighbors^70,71^. For hyperparameter tuning, we employed grid search (see supplemental Figs. 4, 5) with CV as it typically provides reliable results^72–74^.

### Model evaluation

**Fig. 2:**
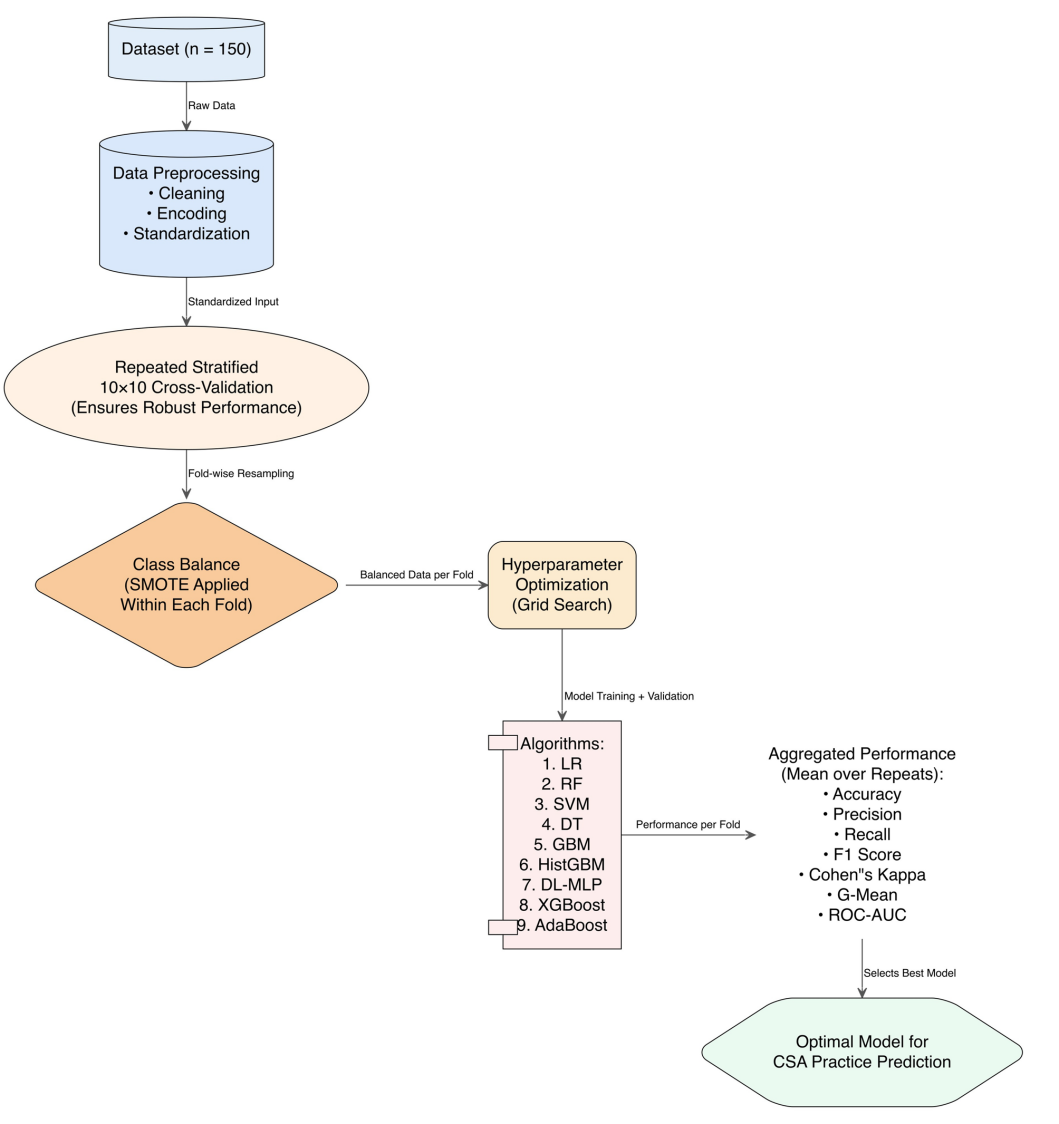
Details view of the selected nine ML framework.

The test dataset was used to evaluate model performance across various metrics such as accuracy, precision, recall, F1 score, area under curve (AUC), precision-recall (PR) curve, Matthews’ correlation coefficient (MCC) and g-mean score^75–79^. The ML approach is presented in Fig. 2.

## Results

In the univariable analysis, we identified several factors significantly associated with adoption of CSA practices, including the following: farm size, farmer education level, experience in mushroom farming (years), cultivation on owned mushroom land, awareness of CSA practices, knowledge of soil quality, access to climate-related information, frequency of extension visits, participation in mushroom training, access to credit, membership of farming groups, off-farm job opportunities, and availability of local storage facilities. The highest proportion of CSA adoption was observed in Savar Paurashava (65.9%), followed by Savar (48.5%) and Banagram (36.2%) (Table 1). Farmers’ education levels showed a significant positive association with CSA adoption. For instance, farmers with no formal education had the lowest adoption rates, whereas those with secondary education or higher had the highest rates (29.2% vs. 58.3%).

**Table 1:** Association between variables and CSA adoption among mushroom farmers.

Variables	CSA practice		*p*-value
	Yes (%)	No (%)	
Farmer age (Years)			0.446
< 40	52.0	48.0	
40+	45.8	54.2	
Area			0.017
Banagram	36.2	63.8	
Savar Paurashava	65.9	34.1	
Savar	48.5	51.5	
Sex			0.923
Male	55.0	45.0	
Female	47.0	53.0	
Educational status			0.006
No education (ref.)	29.2	70.8	
Primary	32.0	68.0	
Secondary+	58.3	41.7	
Mushroom farming experience (Years)			0.020
< 5	56.2	43.8	
5+	36.5	63.5	
Mushroom farming in own land			< 0.001
No	7.3	92.7	
Yes	64.7	35.3	
Knowledge about CSA			< 0.001
No	9.8	90.2	
Yes	68.9	31.1	
Knowledge about temperature			0.054
No	14.3	85.7	
Yes	53.3	46.7	
Internet exposure			0.194
No	60.0	40.0	
Yes	52.5	47.5	
Knowledge about soil quality			0.003
No	33.3	66.7	
Yes	58.3	41.7	
Knowledge about light			0.769
No	47.9	52.1	
Yes	50.5	49.5	
Access market			0.376
No	54.2	45.8	
Yes	46.9	53.1	
Knowledge about relative humidity and ventilation for mushroom cultivation			0.129
No	43.6	56.4	
Yes	55.7	44.3	
Electrical conductivity (EC)			0.830
No	48.5	51.5	
Yes	49.6	50.4	
Access to climatic information			< 0.001
No	39.8	60.2	
Yes	71.4	28.6	
Extension visits			0.002
No	15.8	84.2	
Yes	57.3	42.7	
Contract farming			0.645
No	51.0	49.0	
Yes	49.1	50.9	
Transport availability			0.078
No	48.9	51.1	
Yes	56.5	43.5	
Training about mushroom farming			< 0.001
No (ref.)	23.6	76.4	
Yes	71.8	28.2	
Access credit			0.031
No	45.1	54.9	
Yes	65.7	34.3	
Membership			0.047
No	47.1	52.9	
Yes	70.4	29.4	
Mobile phone			0.055
No	14.3	85.7	
Yes	54.5	45.5	
Off farm job opportunity			< 0.001
No	30.9	69.1	
Yes	64.0	36.0	
Local storage			< 0.001
No	33.3	66.7	
Yes	59.8	40.2	

### Feature selection

**Fig. 3:**
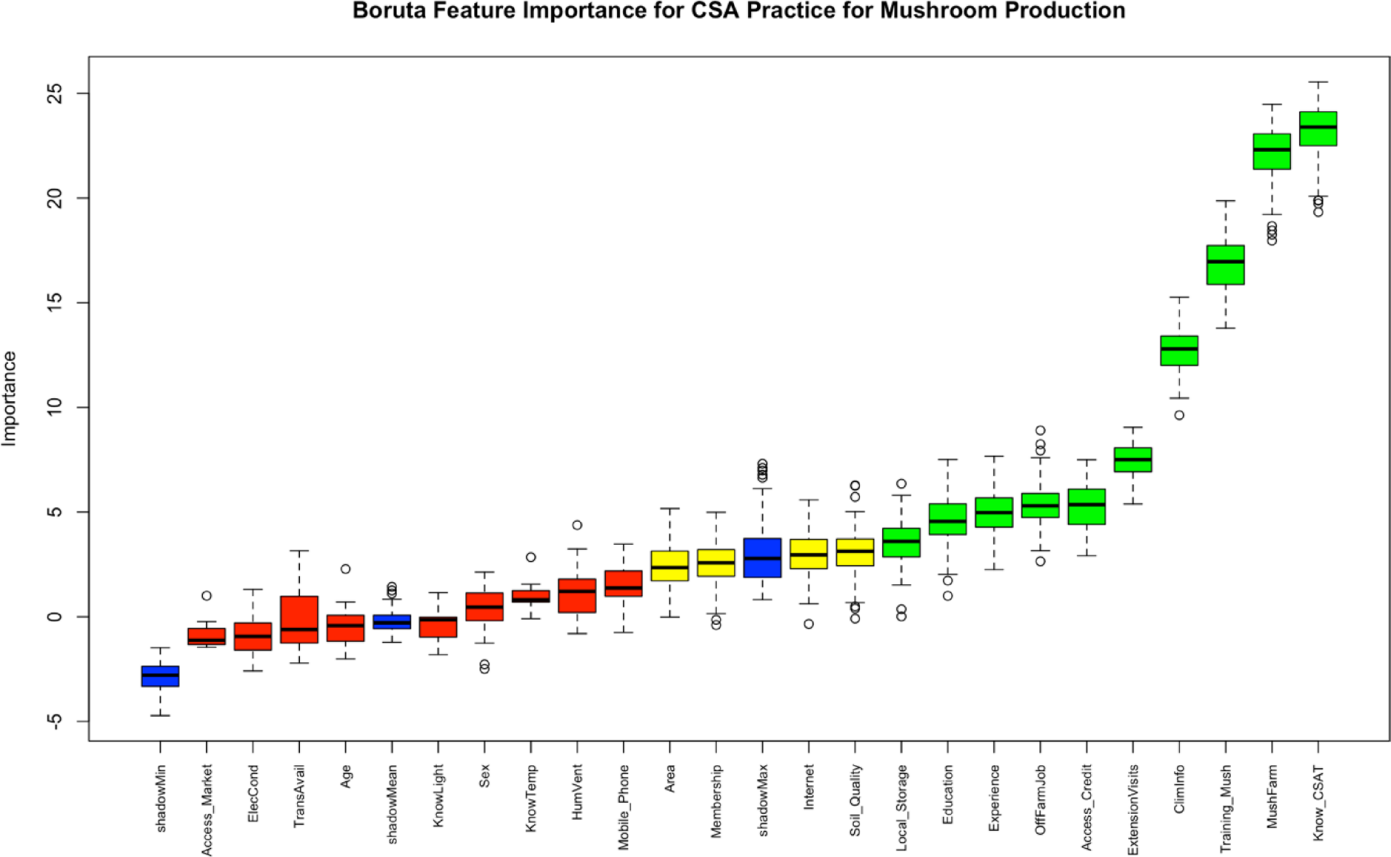
Boruta plot shows the variables importance score for CSA adoption. Blue boxplots represent minimum, average and maximum Z score of a shadow attribute’s. Confirmed and rejected of Z scores qualitied were represented by green and red boxplots. This study does not contain any unimportant characteristics (red box plot).

Figure 3 shows the variable importance determined by Z-score variability from the Boruta algorithm. Top factors associated with CSA practices identified through high importance scores (> 10) included the following: knowledge about CSA practice, owning a mushroom farm, training about mushroom and access to climate information.

**Fig. 4:**
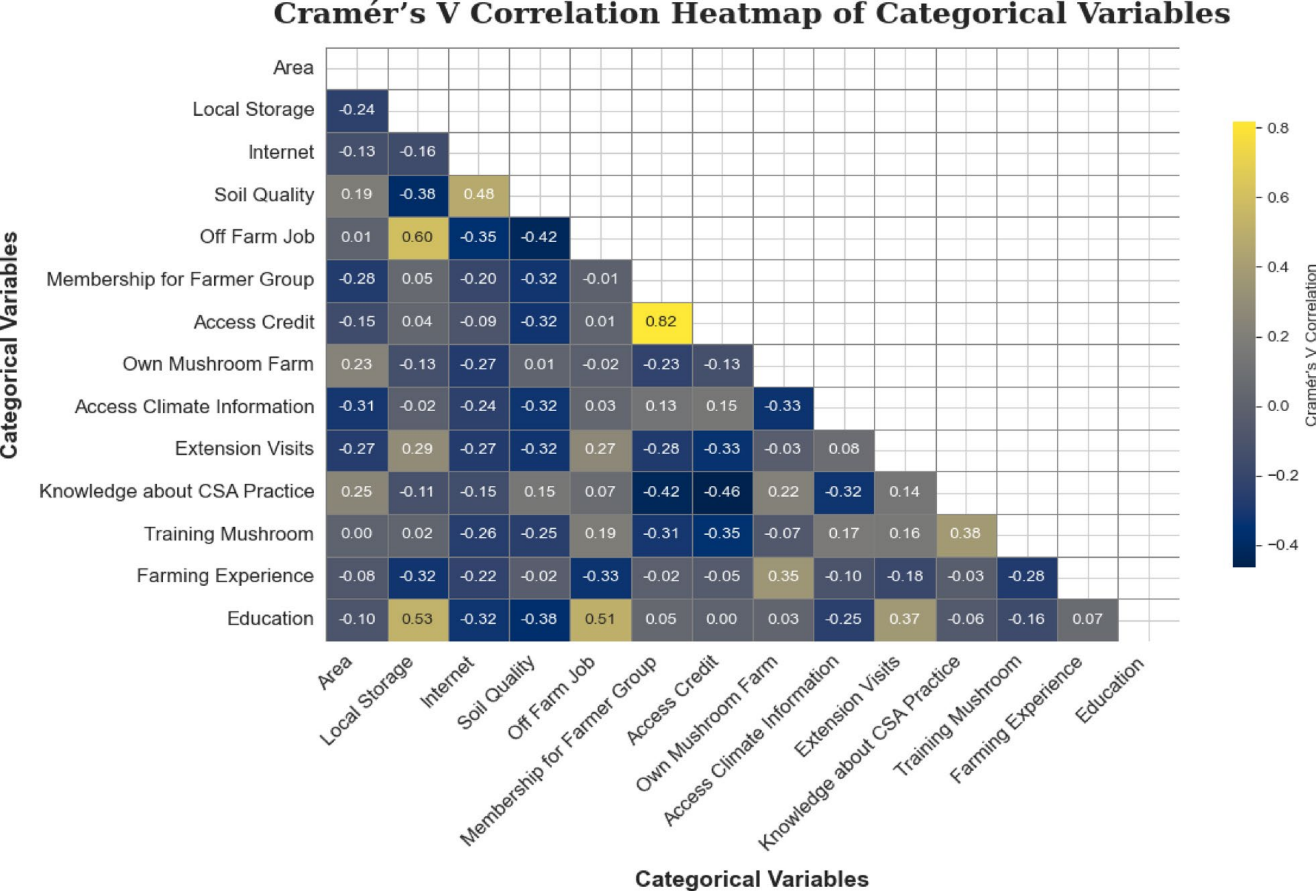
Correlations among categorical features using Cramer’s V correlation coefficient. Cramer’s V scales the Chi−square statistic to a range of 0–1, offering an intuitive measure of association for categorical variables. The vertical axis, with a color gradient from dark blue to yellow, yellow represents positive (upward) correlations among categorical features and darker blue color indicate negative (downward) correlations among categorical.

Based on the Cramér’s V correlation, the ‘membership of farmer group’ variable was excluded due to its strong correlation with ‘access to credit’ (V = 0.82), and the ‘local storage’ variable was removed because of its moderate correlation with ‘off-farm job’ (V = 0.60) (Fig. 4).

### Determinants of CSA practice among mushroom farmers

For convergence diagnostics, all R-hat values were equal to 1, indicating that chains in our Bayesian model have converged and that the parameter estimates were stable (Table 2). Our Bayesian models revealed that factors such as education status, ownership of land for mushroom farming, knowledge of CSA practice, access to climate information, access to credit, mushroom training and secondary education were statistically significantly associated with adoption of CSA practices (Table 2). For a complete description of model fitting, refer to the supplemental materials (Supplemental Figs. 6, 7, 8, 9, 10 and text, page 07).

Farmers with secondary education were 1.53 times more likely to adopt CSA practices than those with no formal education. Farmers who owned land for mushroom farming had 3.68 times higher odds of adopting CSA practices compared to those without land ownership. Additionally, farmers with knowledge of CSA practices were 3.61 times more likely to adopt it than those without such knowledge.

Mushroom training was also associated with CSA adoption. Farmers who received training on mushroom farming had 3.04 times higher odds of adopting these practices compared to their counterparts. Farmers with access to climate information and credit were 2.69 and 2.79 times more likely, respectively, to adopt CSA practices compared to their counterparts.

**Table 2:** Variables associated with adoption of CSA practices among mushroom farmers in Bangladesh as identified by bayesian logistic regression models.

Variables	aOR (95% HDI)	*R*-hat
Area		
Banagram (ref.)	1	
Savar Paurashava	0.48 (0.16–1.43)	1.00
Savar	1.90 (0.69–3.82)	1.00
Educational status		
No education (ref.)	1	
Primary	0.88 (0.24–2.78)	1.00
Secondary	1.53 (1.13–3.20)	1.00
Mushroom farming experience (Years)		
< 5	1	
5+	0.75 (0.94–1.50)	1.00
Extension visits		
No (ref.)	1	
Yes	1.32 (0.60–3.26)	1.00
Own land mushroom farming		
No (ref.)	1	
Yes	3.68 (1.76–7.34)	1.00
Knowledge about CSA practice		
No (ref.)	1	
Yes	3.61 (2.69–7.68)	1.00
Training about mushroom		
No (ref.)	1	
Yes	3.04 (1.62–5.64)	1.00
Access about climate information		
No (ref.)	1	
Yes	2.69 (1.33–5.51)	1.00
Access credit		
No (ref.)	1	
Yes	2.79 (1.06–3.46)	1.00
Knowledge about soil quality		
No (ref.)	1	
Yes	0.54 (0.21–1.41)	1.00
Internet		
No (ref.)	1	
Yes	0.76 (0.28–2.11)	1.00
Off farm job opportunity		
No (ref.)	1	
Yes	1.63 (0.78–2.78)	1.00
Model fitness		
WAIC		
ELPD-WAIC	-59.2	
P-WAIC	4.9	
WAIC	118.4	
LOO-CV		
ELPD-LOO	-59.2	
P-LOO	5.0	
LOOIC	118.5	
Prediction performance		
Accuracy	0.875	
AUC	0.967	

ref. = Reference category.

### Evaluation of prediction performance of ML models

**Fig. 5:**
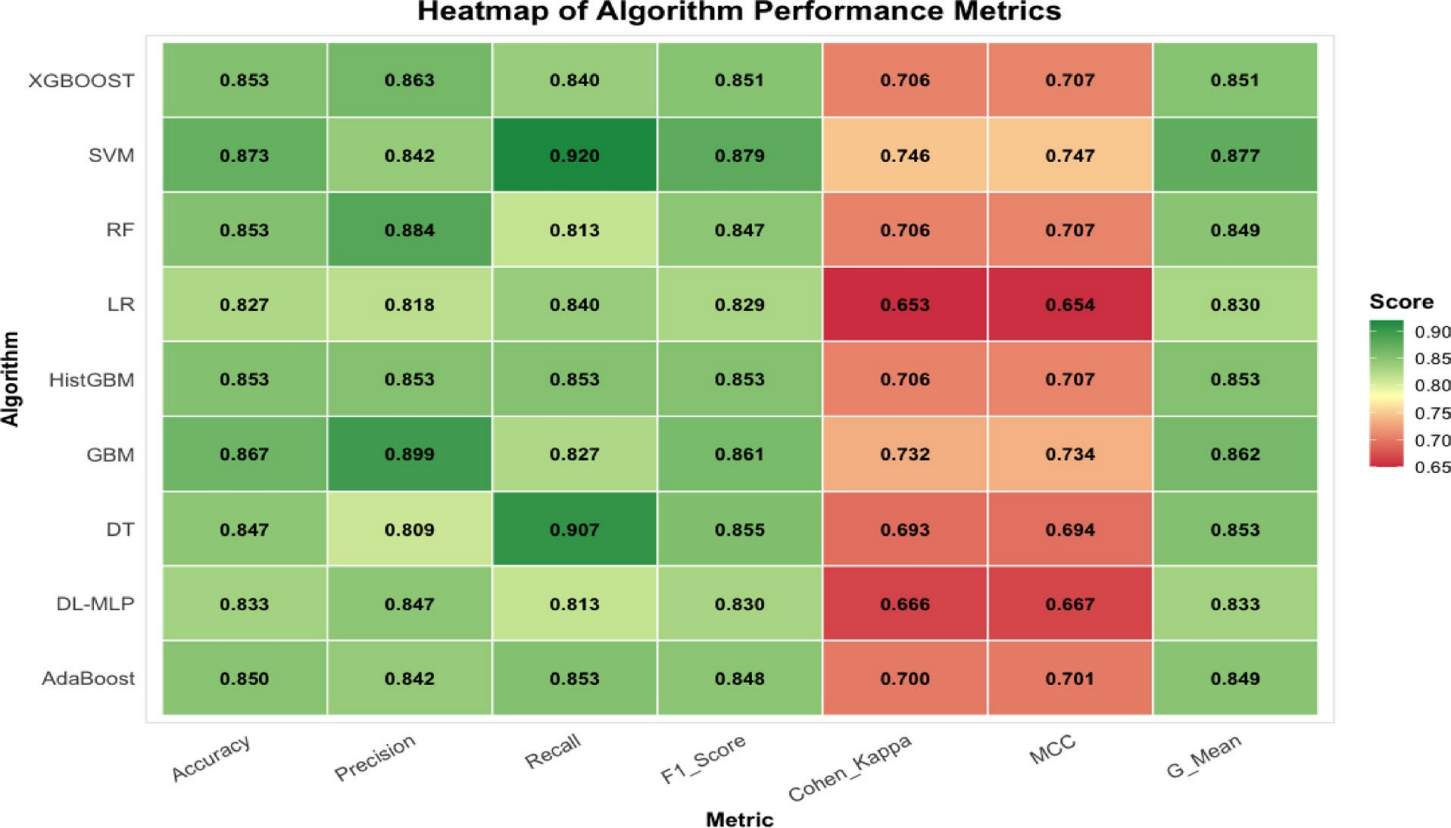
Comparative heatmap of ML performance across multiple metrics (accuracy, precision, recall, F1 score, Cohen-Kappa, MCC and g-mean score).

**Fig. 6:**
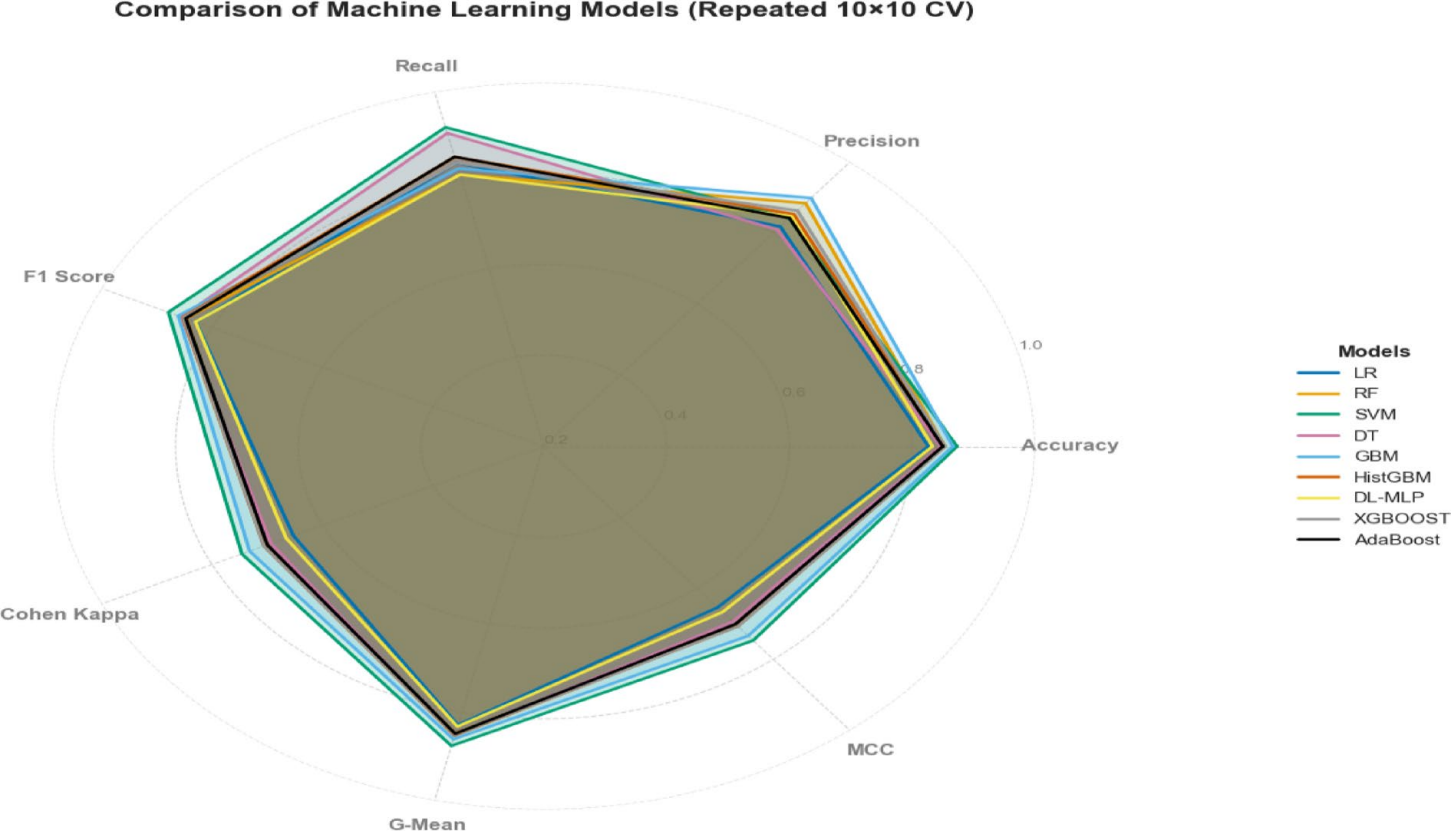
The radar plot illustrates a comparison of the performance of the selected nine algorithms for predicting CSA practice among mushroom farmers. Each model is represented by a separate axis, with values ranging from 0 to 92% for each metric, all converging at the center of the chart. Radar plots are particularly effective for identifying outliers and areas of overlap.

The confusion matrix for classifiers can be seen in Supplemental Fig. 11. Among nine classifiers, the SVM algorithm achieved the best performance for predicting adoption of CSA practices, with an accuracy of 87.3%, Recall of 92%, F1 score of 87.9%, Cohen’s Kappa of 74.6%, MCC of 74.7% and g-mean score of 87.7% (Fig. 5). However, in terms of precision, GBM had greater performance achieving 89.9% precision. Similarly, GBM also shows the second highest performance for, Cohen Kappa (73.2%), MCC (73.4%) and g-mean score (86.2%). The Cohen’s Kappa value for the SVM classifier was 0.746 indicating strong reliability with an agreement level above 74.6% followed by GBM (73.2%) and RF (70.6%; Fig. 6).

**Fig. 7:**
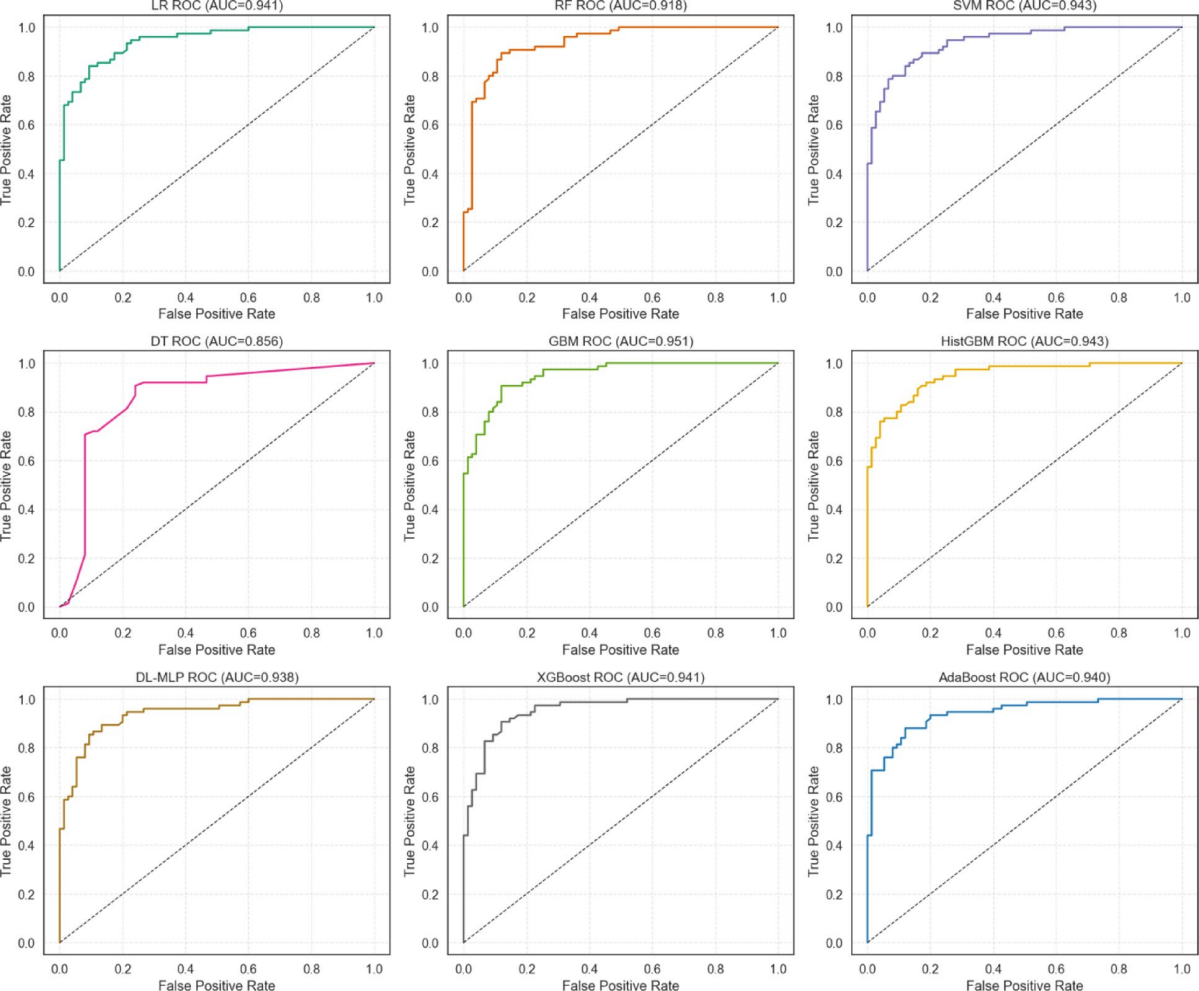
A comparison of ROC curves and AUC values shows the predict CSA practice for mushroom production. These comparisons were made for nine different ML algorithms using SMOTE, repeated stratified 10-fold CV with hyperparameter. For ROC curve, which compares true positive rate (sensitivity against the false positive rate across different thresholds to evaluate algorithm performance. The AUC, ranging from 0 (random guessing) to 1 (excellent performance), quantifies overall discriminative ability.

AUC values were calculated for all selected algorithms (Fig. 7). The highest AUC values were estimated for GBM (AUC = 0.951), followed by SVM (AUC = 0.943), HistGBM (AUC = 0.943), XGBoost (AUC = 0.941), LR (AUC = 0.941), AdaBoost (AUC = 0.940), DL-MLP (AUC = 0.938), RF (AUC = 0.918), and DT (AUC = 0.856).

PR, distribution of accuracy and g-mean scores, decision curve and learning according to CV analysis can be located in the supplemental files (Figs. 12, 13 and 14). In general, the GBM algorithm (average precision (AP) = 0.954) demonstrated the highest discriminatory ability among all tested ML algorithms.

### Feature importance

**Fig. 8:**
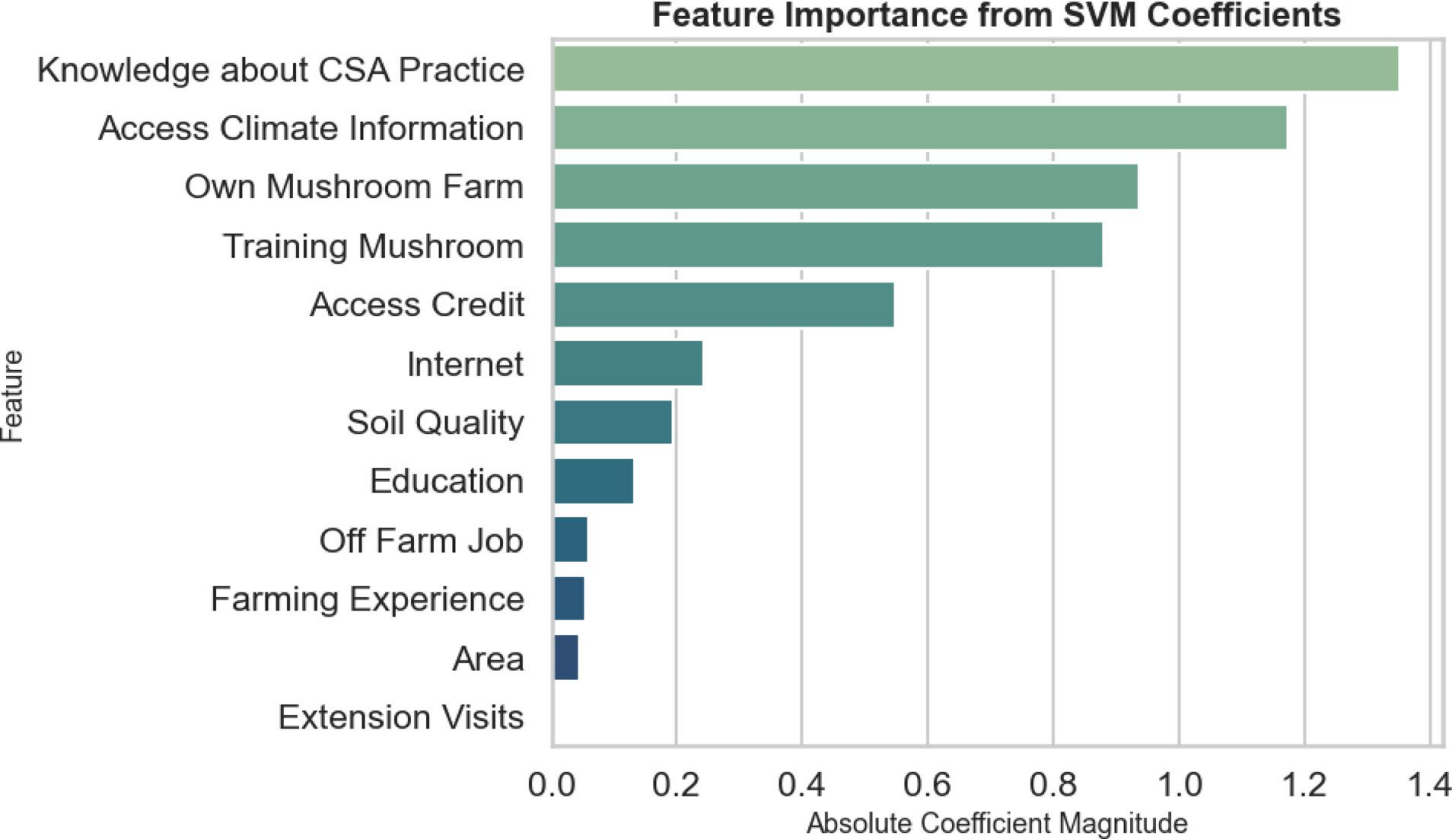
Feature importance for predict CSA practice based on SVM algorithm. The length of each bar represents the mean importance of the feature.

Knowledge about CSA practice was the most influential factor for predicting the adoption of CSA practices for mushroom cultivation followed by access climate information, ownership of farming land, previous training in mushroom farming, and to access credit (Fig. 8).

## Discussion

Here we investigated factors that were associated with adoption of CSA practices in mushroom farming in Bangladesh. Bayesian analysis revealed that farmer education, farm ownership, CSA knowledge, mushroom training, climate information access, and credit availability were significant and potential factors influencing CSA practice. In Ghana, smallholder maize farmers adopt CSA practices based on factors like education, farmer group membership, land access, market availability, and production challenges^80^.

Educational background and farming experience were significantly associated with the adoption of CSA practices. Farmers with secondary or higher education are more likely to adopt CSA, as education enhances comprehension of new technologies and engagement with extension services^13,81,82^. Higher education enhances farmers’ access to new technology information, increasing their likelihood of adoption^83^. Farmers with education, drought experience, and awareness of rainfall changes were more likely to adopt CSA practices^84^. Similar to prior studies suggesting resistance among experienced farmers, our findings indicate that those with five or more years of mushroom farming experience are less receptive to CSA^82,85^.

Farm ownership emerged as a critical adoption determinant, underscoring the role of tenure security in facilitating long-term sustainability investments. Secure landholders are more inclined to adopt CSA practice due to greater control over land-use decisions and investment planning^85,86^. Similarly, knowledge and training significantly enhance adoption, aligning with research highlighting the role of awareness and technical exposure in shaping farmer behavior^82,87^.

Access to climate information was another key factor, supporting that real-time weather forecasts enable informed decision-making and risk management^88,89^. Access to credit significantly increases the likelihood of adopting CSA practice compared to those without credit access. Farmers with more savings and credit access are more likely to adopt CSA practices^86^, as financial resources mitigate liquidity constraints and enable investment^81,82^. Additionally, perceived benefits, such as yield stability and cost savings, strongly drive adoption, reinforcing the economic rationality of farmers in technology uptake^90,91^.

Interestingly, traditional extension services, mobile phone usage, and off-farm employment were not significantly linked to the adoption of CSA practices in our models. This suggests that conventional extension approaches may be insufficient without interactive, context-specific engagement^92^. While digital technologies are often emphasized in knowledge transfer, their effectiveness likely depends on service design and alignment with farmers’ learning preferences. The non-significant effect of off-farm employment challenges the notion that income diversification alone enhances adoption, indicating that farmers may prioritize direct on-farm investments over external income sources^83^.

This study investigates ML approaches to predict the adoption of CSA practices in mushroom growing. In terms evaluation metrics of ML, SVM and GBM shows slightly outperformed other algorithms for predicting the adoption of CSA practices in mushroom farming. CNNs are the most widely algorithms in mushroom farming studies, followed by k-nearest neighbors and NB^26^. YOLOv5 is utilized to identify ready-to-harvest mushrooms in greenhouses, with an F1-score of up to 76.5% and an accuracy of up to 70% in the final stage of mushroom growth, despite the complexity of used photos^93^.

Both Bayesian and ML models identified knowledge about CSA practices, access to climate information, training on mushroom cultivation, ownership of land for mushroom farming, access to credit and education as significant predictors of CSA adoption. However, ML models additionally highlighted internet and knowledge about soil quality as an important factor influencing CSA uptake, which likely reflects ML’s strength in uncovering complex, nonlinear patterns^94^that may be less apparent in traditional analyses.

Our findings have strong potential for guiding agricultural decisions. Understanding factors behind CSA adoption can support policymakers develop focused strategies to stimulate their adoption, such as training programs. Additionally, financial incentives related to land access, as well as improved access to climatic information and CSA resources via the internet, could foster broader participation in CSA initiatives. Nevertheless, this study has limitations. This is a cross-sectional study, which means we cannot establish cause and effect. The binary ‘adoption’ variable, which captures whether respondents adopted at least one CSA practice, does not account for the intensity, diversity, or combined effects of multiple practices. Additionally, the study only covers a single area in Bangladesh. Moreover, while this study focuses exclusively on quantitative analysis, future research could incorporate qualitative approaches (e.g., interviews or focus group discussions) to explore farmers’ perceptions and motivations, providing a deeper contextual understanding of CSA adoption behavior.

## Conclusion

Several factors significantly influenced the adoption of CSA practices in mushroom farming in Bangladesh. Farmer education, farm ownership, knowledge about CSA practices, training on mushroom farming, access to climate information, and access to credit were the most influential predictors of the adoption of CSA practices in. Among the nine ML models tested, SVM and GBM outperformed others. ML algorithms also highlighted internet, knowledge about soil quality as relevant features. Based on our findings, farmer education and training related mushroom farming, access to climate information and credit, and strengthening land tenure security will be key elements influencing adoption rates.

## Supplementary Information

Below is the link to the electronic supplementary material.

Supplementary Material 1

## Data Availability

The datasets used and/or analyzed during the current study are available from the corresponding author on reasonable request.
