# Supplementary material for: Determinants of adoption of climate-smart agriculture (CSA) practices in mushroom farming in Bangladesh
Source: Sci Rep. 2026 Feb 19;16:9942. doi: 10.1038/s41598-026-39761-4 (PMC13022477; doi:10.1038/s41598-026-39761-4)
Supplement: Supplementary file 1 — Supplementary Material 1 [file 41598_2026_39761_MOESM1_ESM.docx]

**Supplemental**


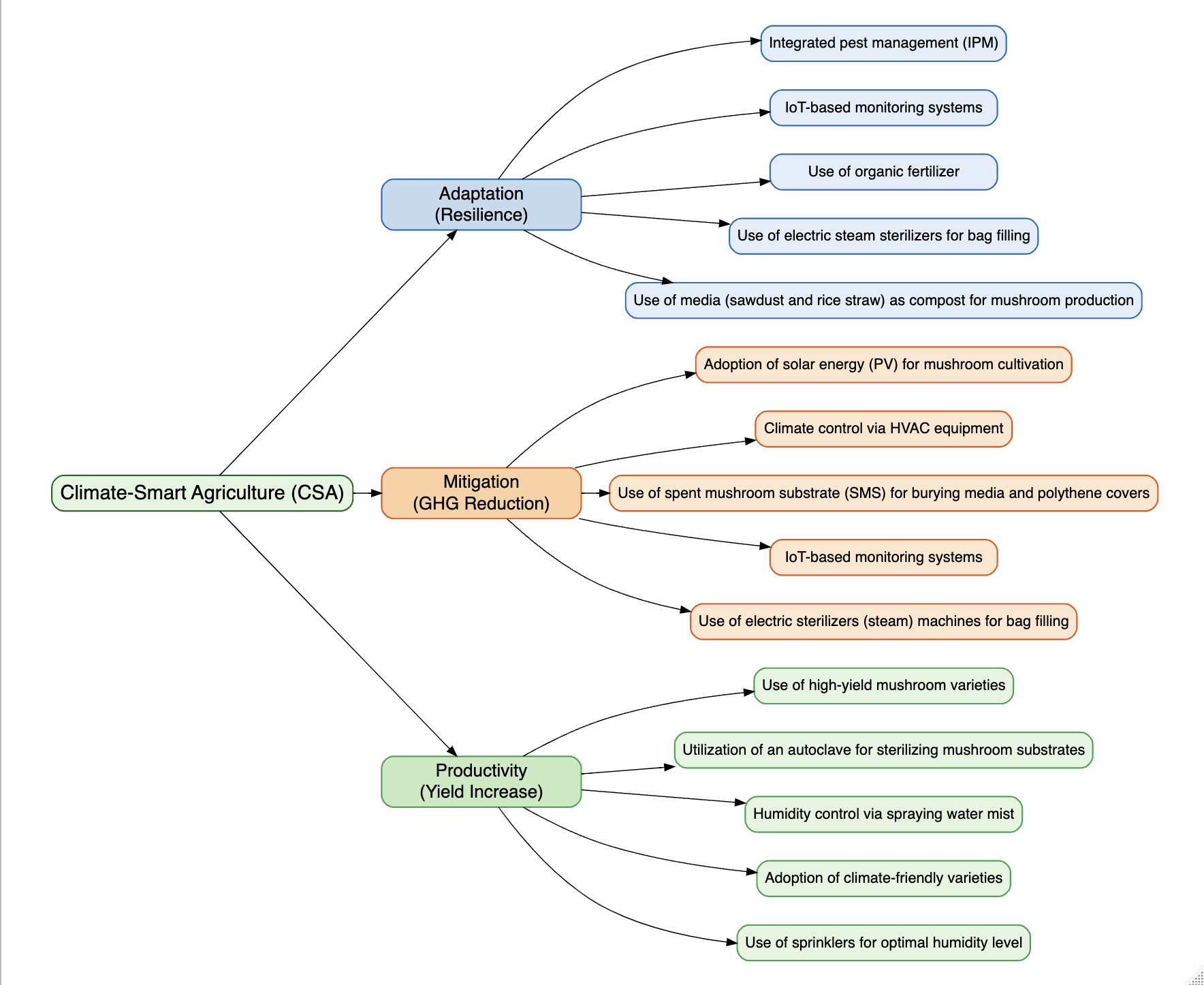


Figure 1. Conceptual framework linking mushroom-related practices to the core pillars of CSA.

Table 1. Frequency and percentage of adoption of individual and pillar-level CSA practices among mushroom farmers

|  | Practice | n(%) |
| --- | --- | --- |
| **Individual^a^** |  |  |
|  | Utilization of an autoclave for sterilizing mushroom substrates | 10 (6.7%) |
|  | Adoption of climate-friendly varieties (oyster mushrooms, and king oyster) | 59 (39.3%) |
|  | Use of high yielding mushroom varieties (*Pleurotus spp.* And *Pleurotus eryngii*), | 67 (44.7%) |
|  | Utilization of IOT-based monitoring and control systems | 6 (4.0%) |
|  | Use of organic fertilizer | 65 (43.3%) |
|  | Climate control via heating, ventilation, and air conditioning (HVAC) equipment | 8 (5.3%) |
|  | Adoption of solar energy (PV) for mushroom cultivation | 16 (10.7%) |
|  | Use of electric sterilizers (steam) machines for bag filling | 8 (5.3%) |
|  | Humidity control via spraying water mist using spray | 20 (13.3%) |
|  | Utilization of integrated pest management (IPM) practices | 47 (31.3%) |
|  | Use of sprinklers for optimal humidity level | 22 (14.7%) |
|  | Use of media (sawdust and rice straw) as compost for mushroom production | 58 (38.7%) |
|  | Use of spent mushroom substrate (SMS) as an alternative method for burying media and polythene covers | 46 (30.7%) |
| **Pillar** | | |
|  | Adaptation | 184 (42.6%) |
|  | Mitigation | 70 (16.2%) |
|  | Productivity | 178 (41.2%) |

^a^Multiple response


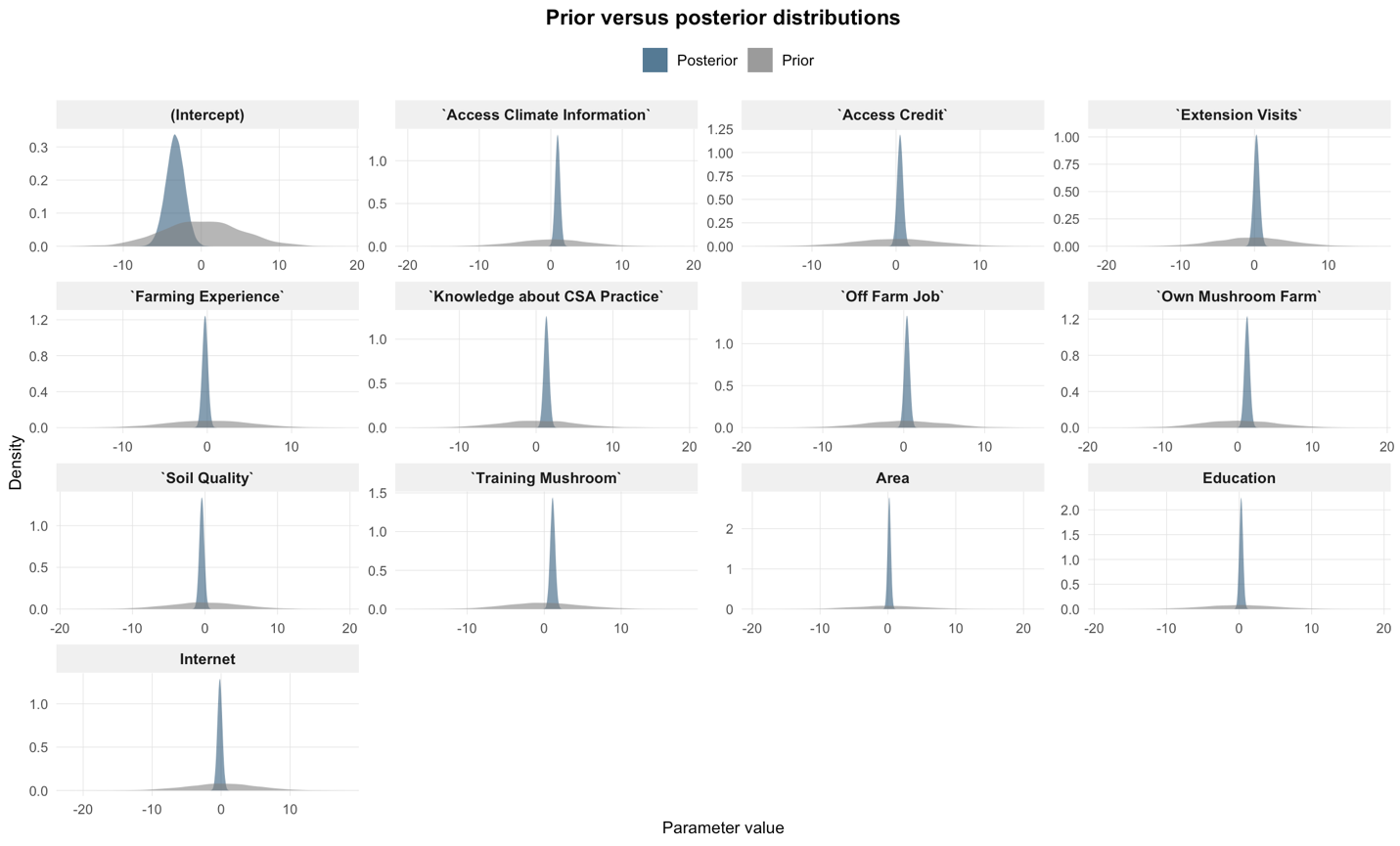
Figure 2: Prior and posterior distributions of parameters influencing CSA practice


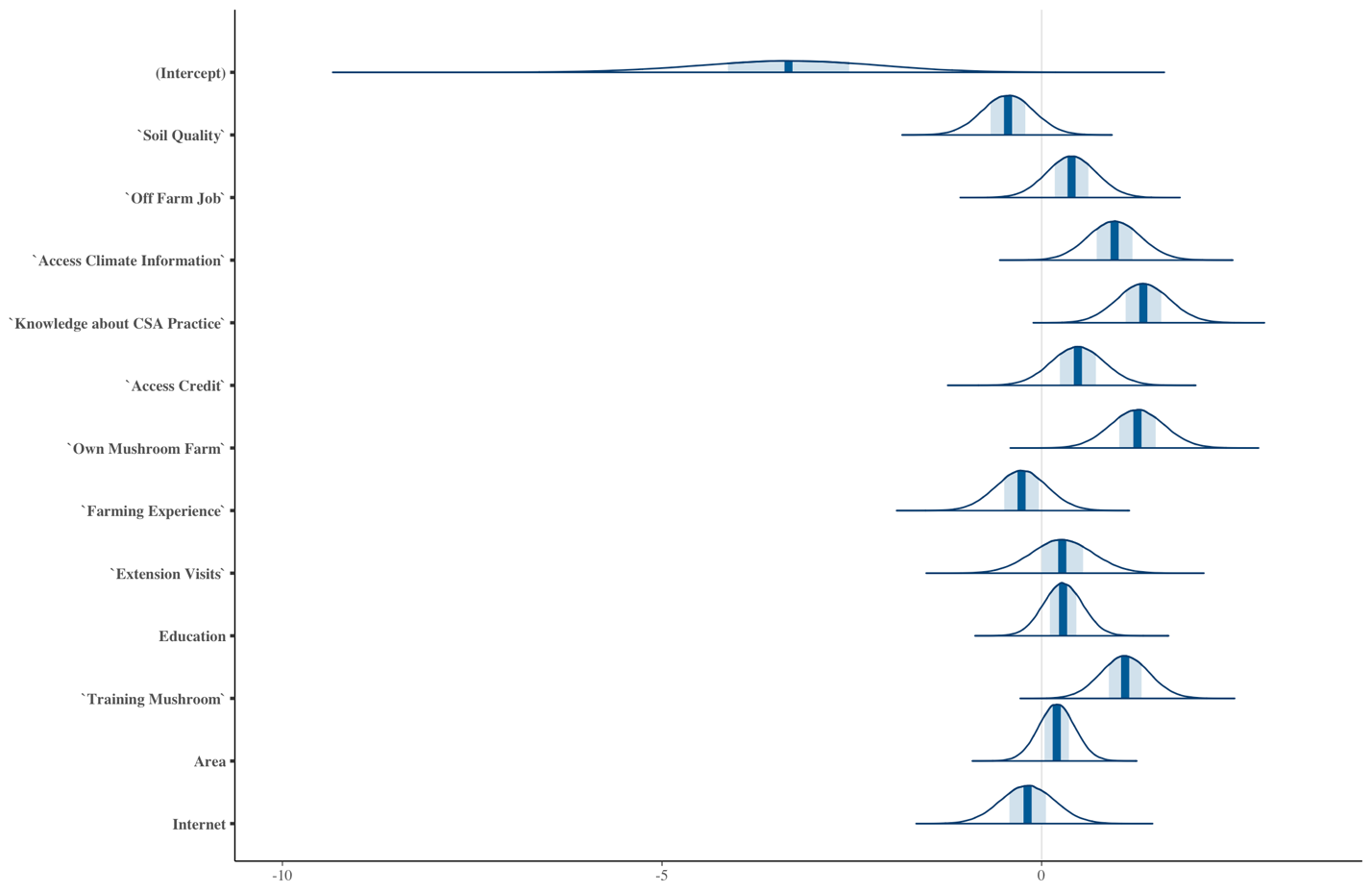


Figure 3. Posterior Distributions of Parameter Estimates in a Bayesian Model of CSA practice


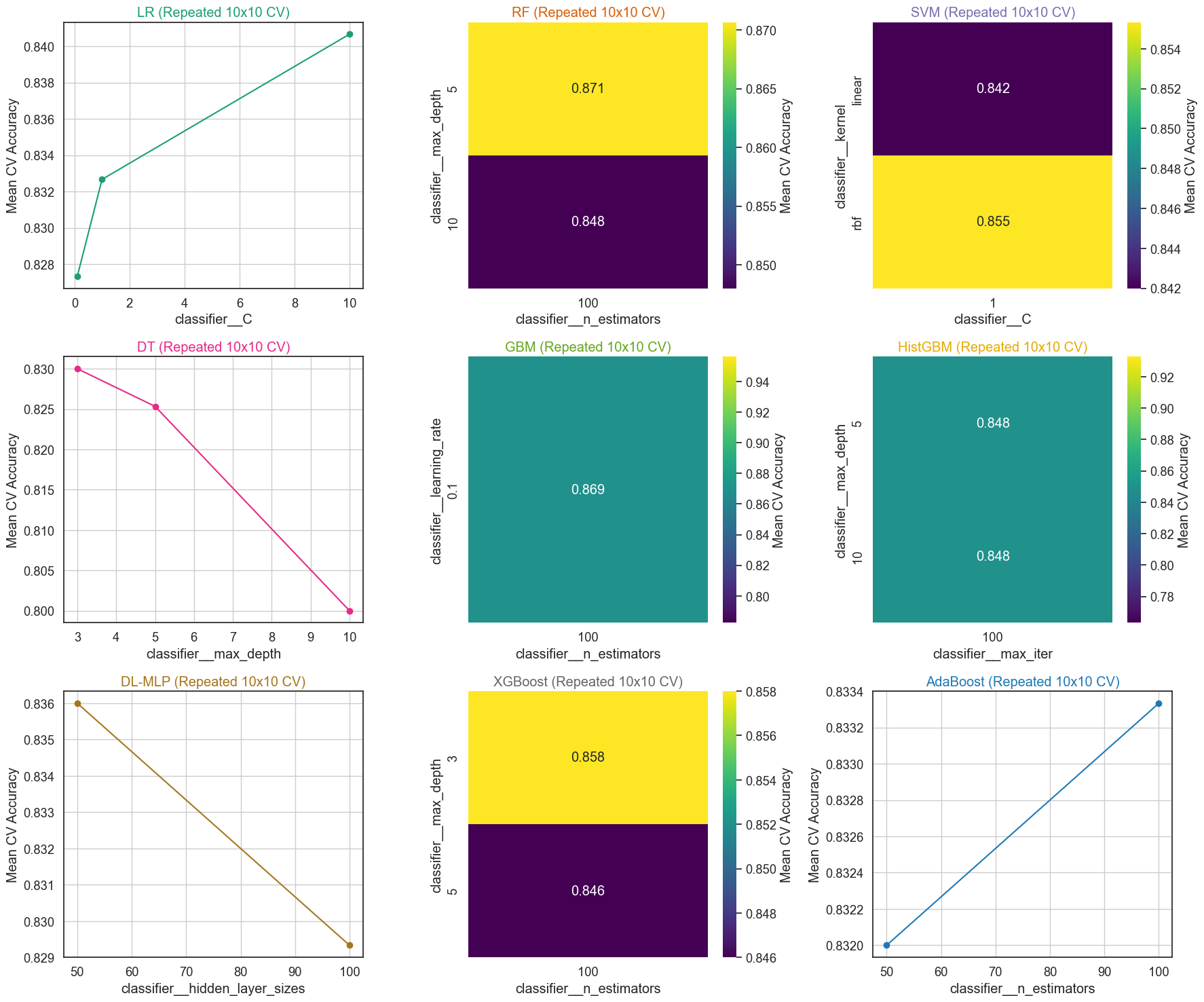


Figure 4. **Hyperparameter Optimization Results Showing Mean CV Accuracy for Each ML Classifier (Repeated Stratified 10×10 CV)**


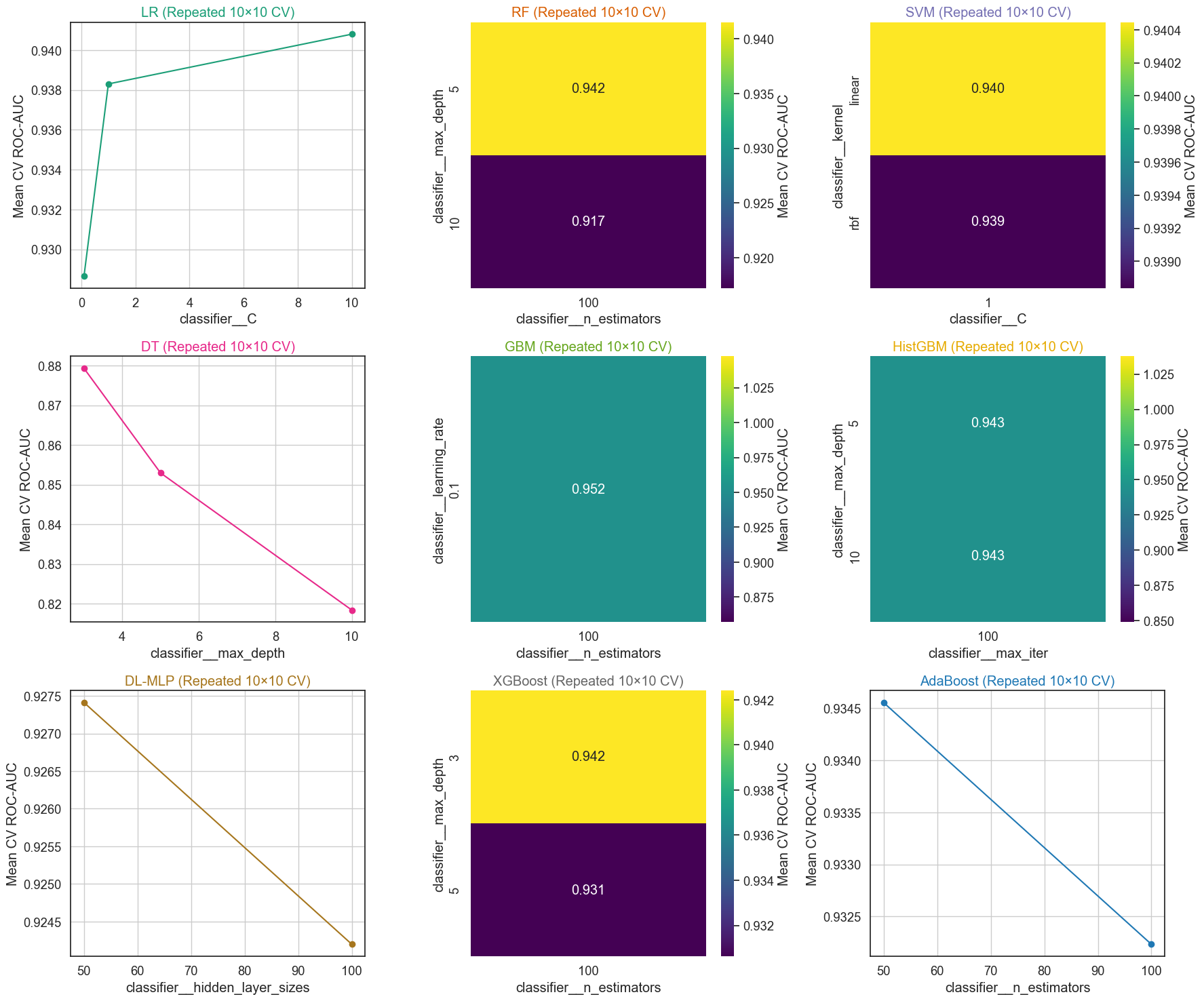


Figure 5. **Hyperparameter Optimization Results Showing Mean CV ROC-AUC for Each ML Classifier (Repeated Stratified 10×10 CV)**

**Description of model fitting,**

Autocorrelation plots for parameters influencing CSA practice in mushroom farming show the autocorrelation function up to 200 lags. The rapid decay to zero indicates increased sample independence over time, suggesting good mixing and convergence of the HMC algorithm, yielding reliable parameter estimates (see supplemental Figure 6). The sampling paths (traces) from the four chains plots indicate that the Bayesian logistic regression model has achieved well HMC convergence and mixing, with stable posterior estimates for the parameters (supplemental Figure 7). The $\hat{R}=1$ further confirms that the chains have fully converged and are well-mixed (see supplemental Figure 8).

The pair plot presents the marginal distributions and bivariate relationships among key selected independent variables. The diagonal panels display histograms that reveal the distributional characteristics of each variable, such as skewness and spread. The off-diagonal scatter plots illustrate pairwise associations, with density contours highlighting regions of high data concentration (supplemental Figure 9).

The Bayesian logistic regression model showed WAIC and LOO-CV values of -59.2 respectively. For its predictive performance, the model achieved a predictive accuracy of 0.875, sensitivity of 0.828, specificity of 0.914, precision of 923 and an F1 score of 0.874. Additionally, the model's AUC was 96.7 percent (supplemental Figure 10).


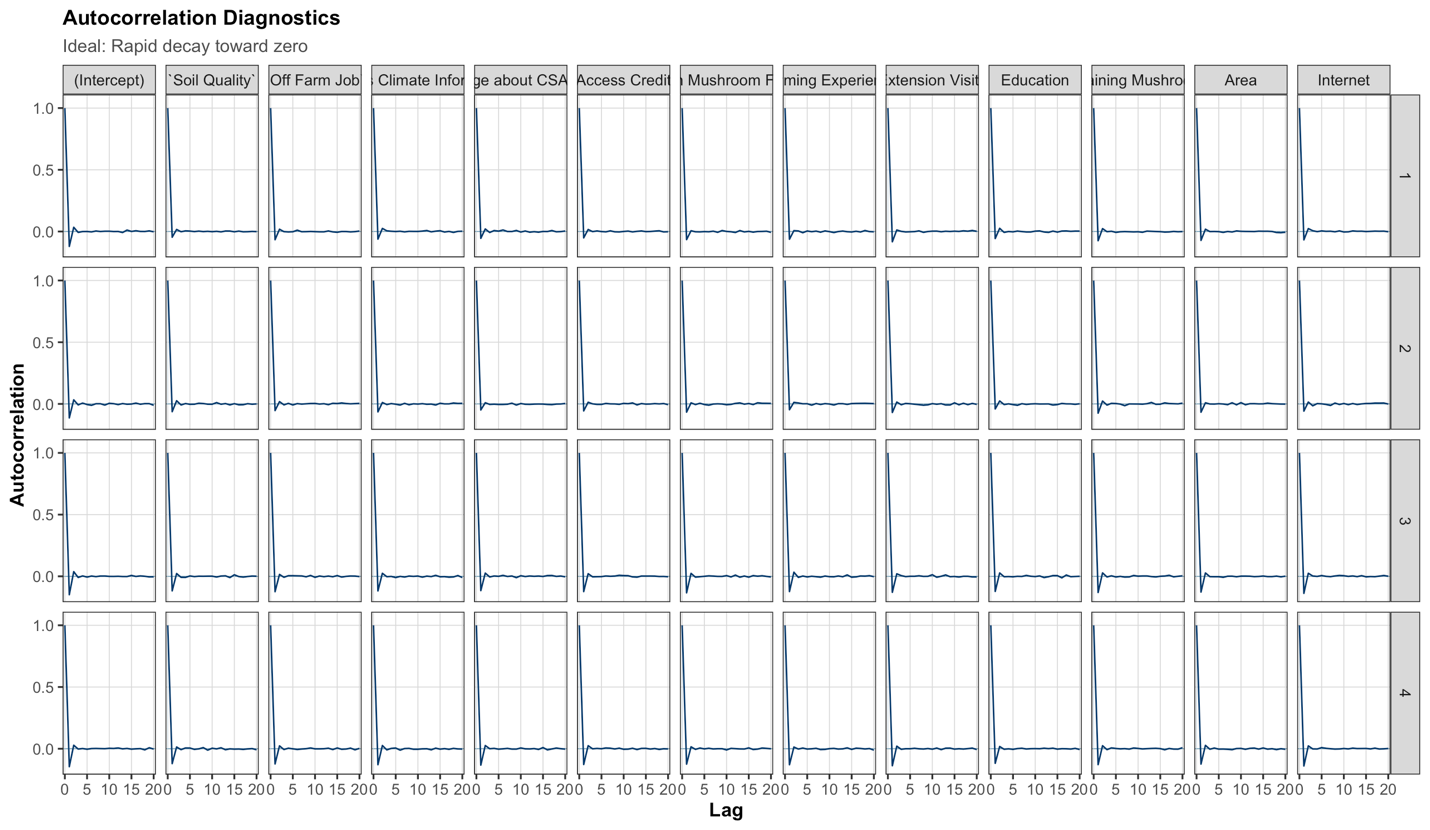


Figure 6. Autocorrelation Analysis of Factors Related to CSA Practices


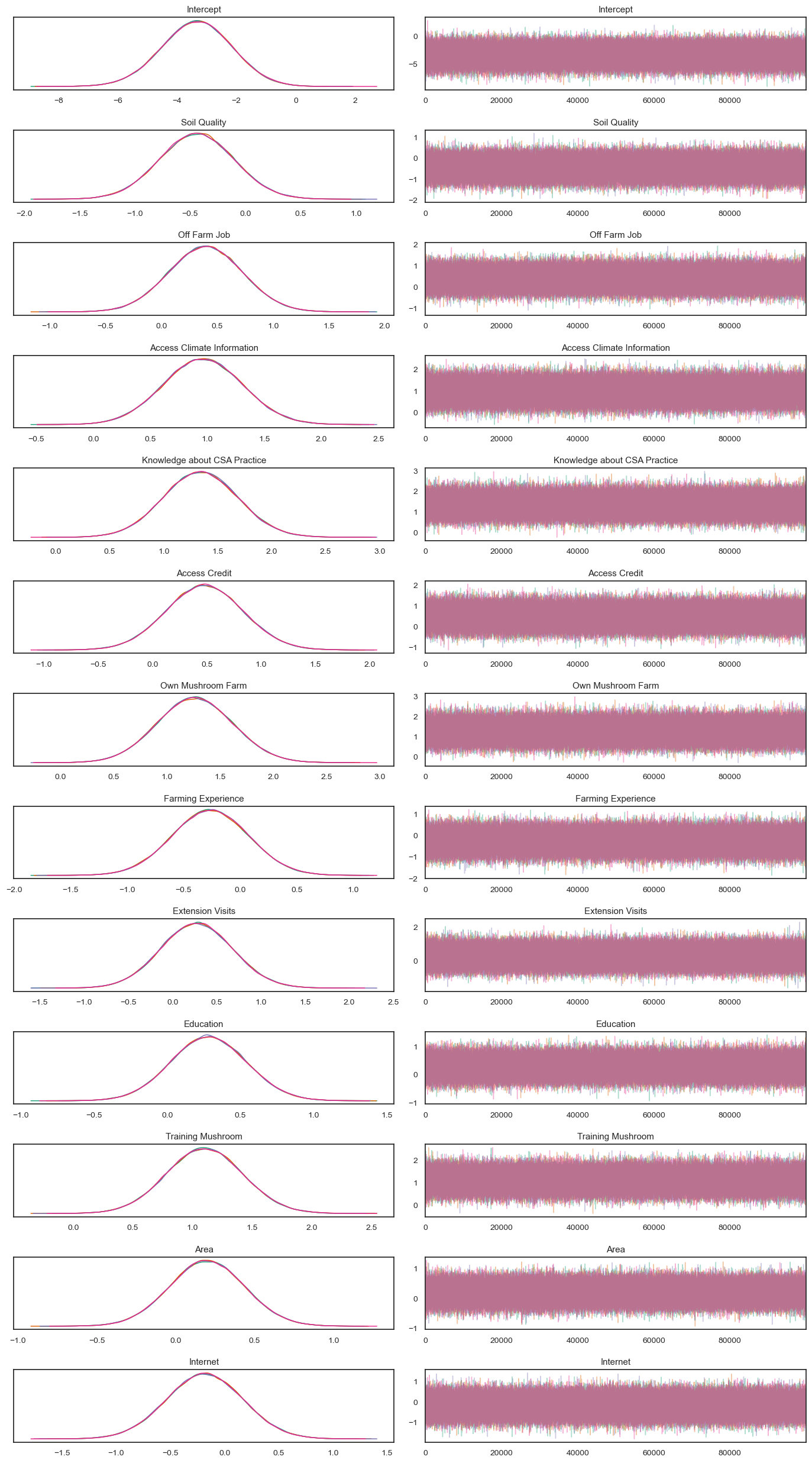


Figure 7. Kernel density estimated shows the marginal posterior distribution for all model parameters (left panel) and Trace plots of HMC simulation for each variable in the Bayesian logistic regression model and which are used to assess parameter convergence across four chains (right panel).


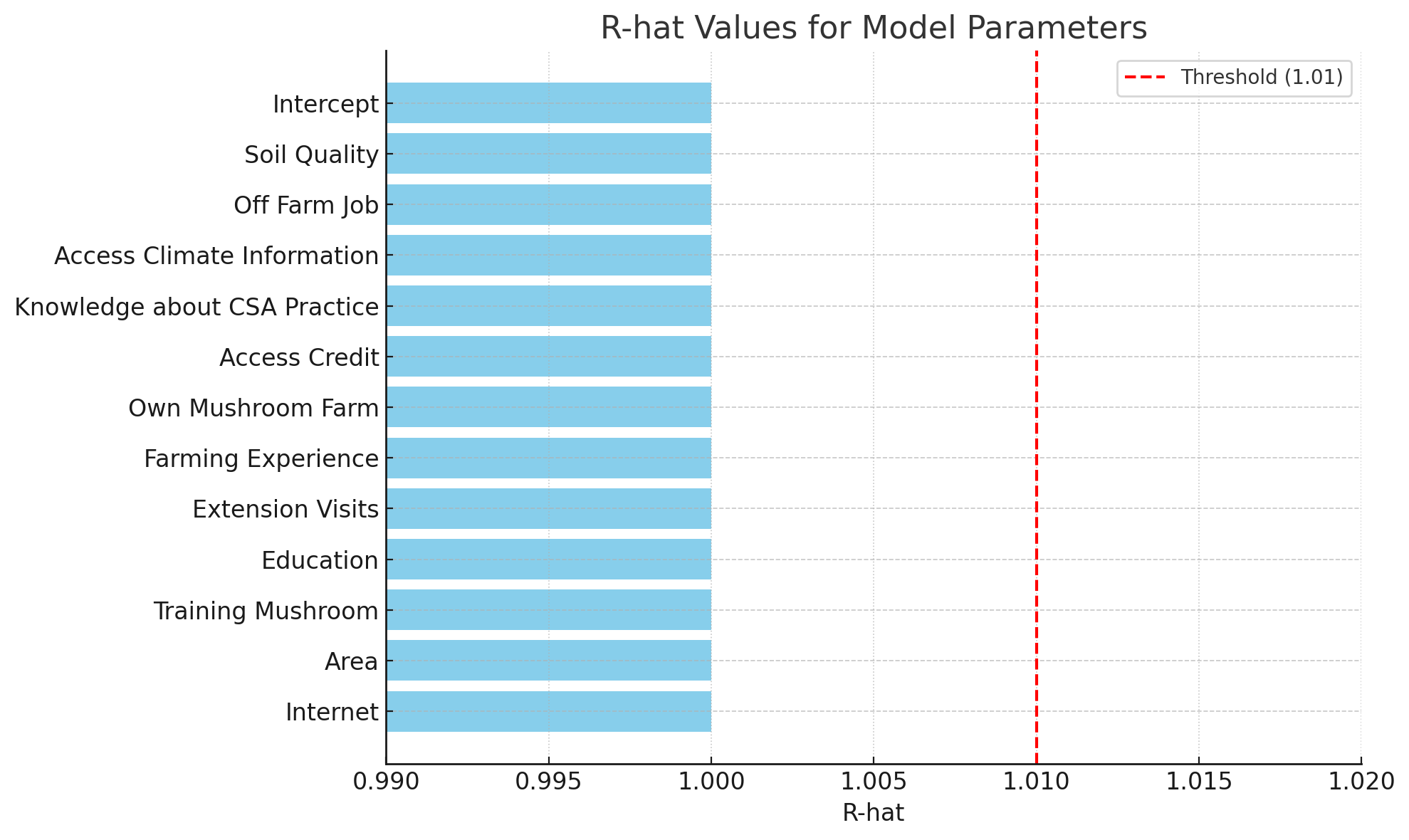


Figure 8. Gelman-Rubin Diagnostic by using R-hat

^
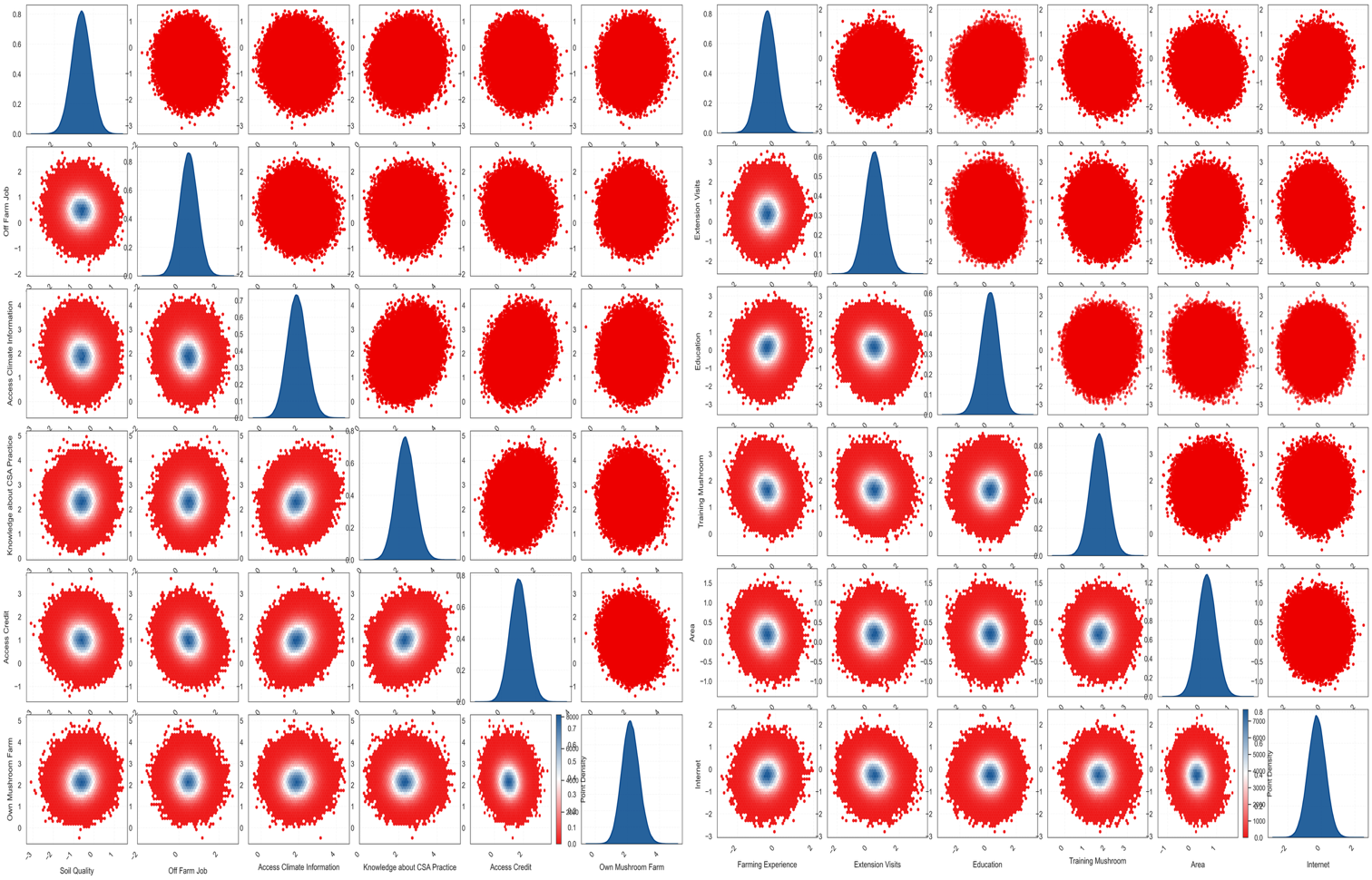
^

Figure 9. Pair plot with hexbin and marginal histograms can be particularly useful for visualizing the posterior distributions and pairwise relationships between parameters in a Bayesian model.

**
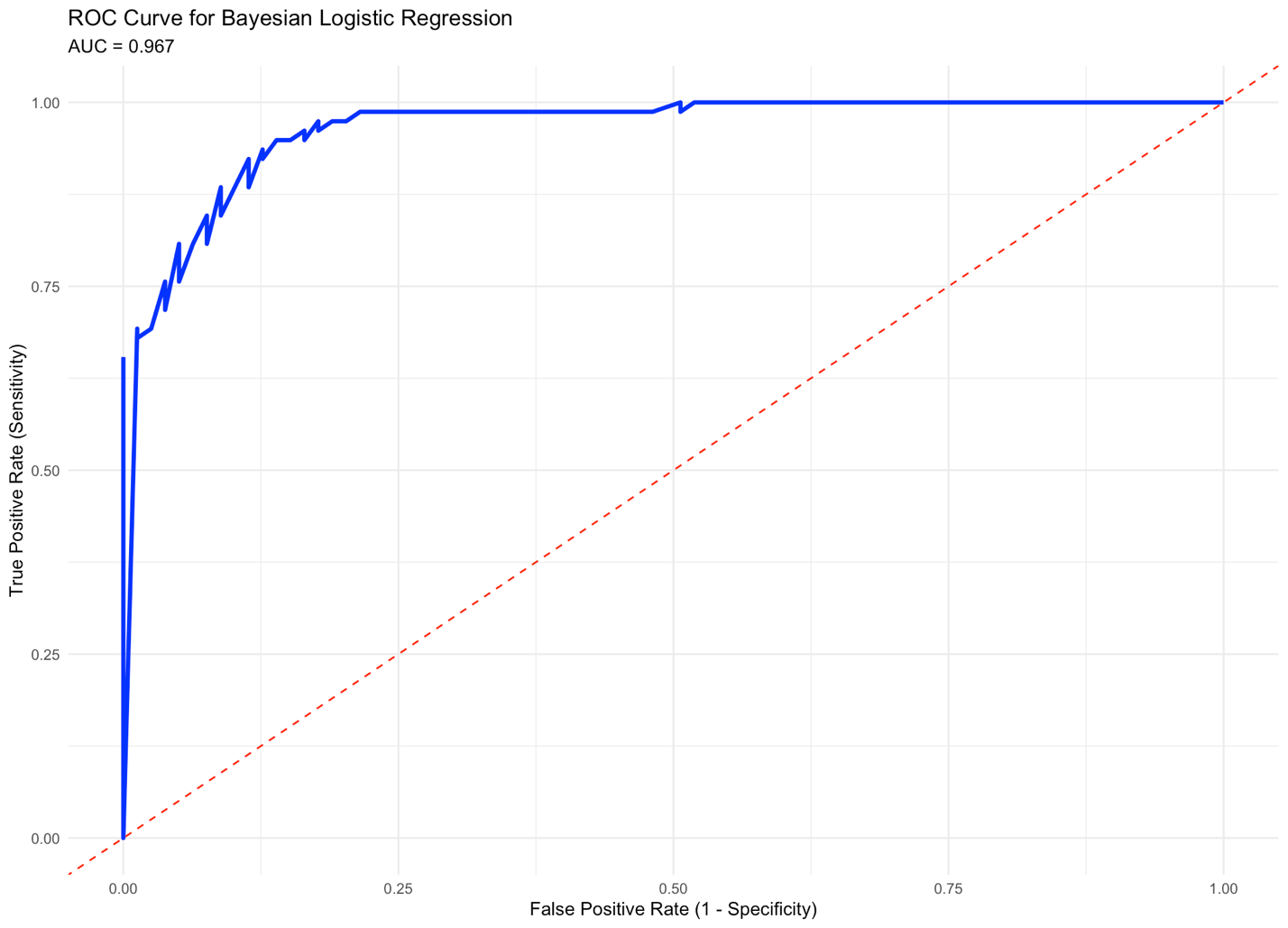
**

Figure 10. ROC curve for Bayesian Logistic Regression


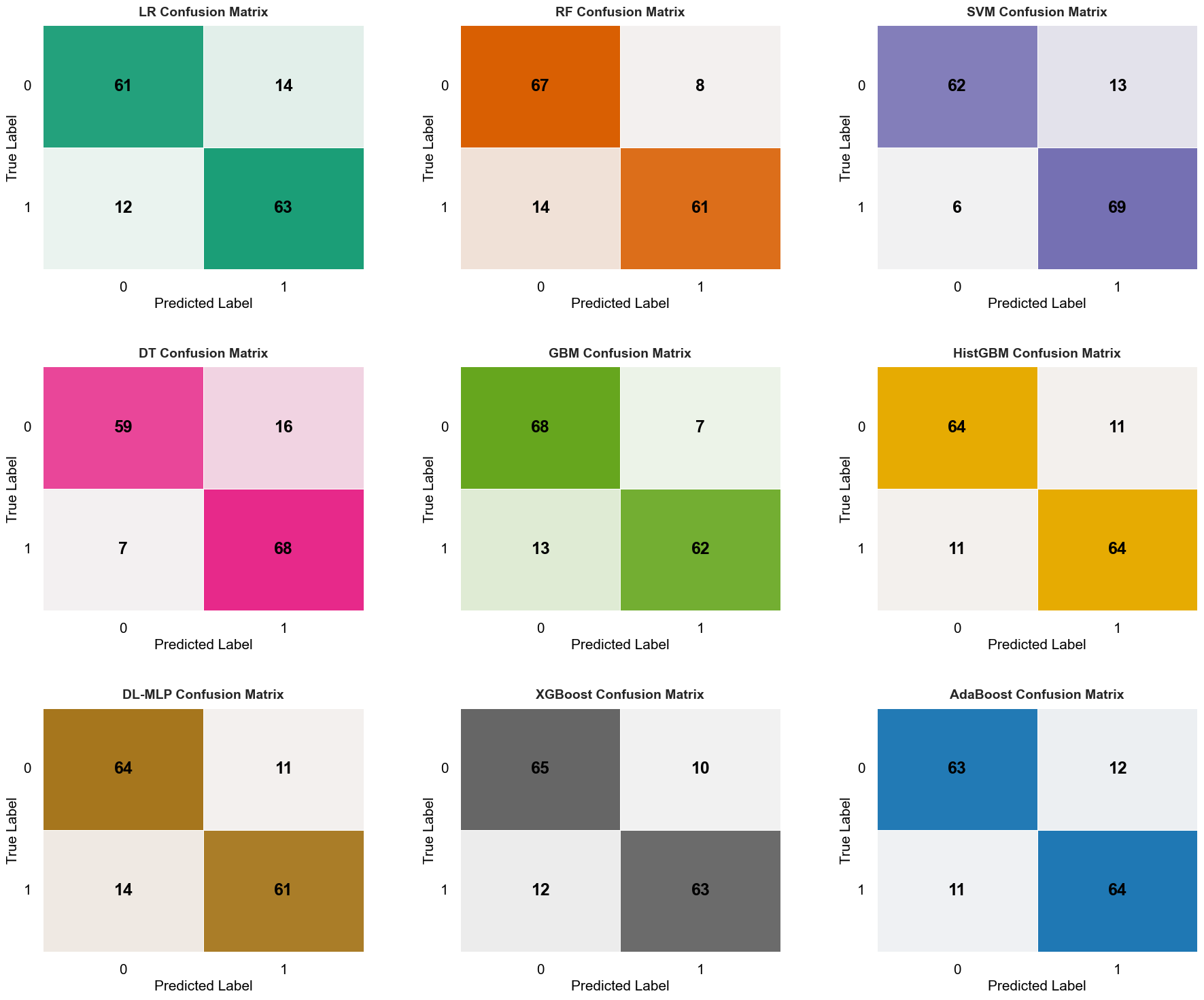


Figure 11. Confusion Matrix among nine classifiers

In terms of the precision-recall curve, among all models, the GBM (AP=0.954) was achieved the precision (AP), followed by HistGBM (AP=0.950), AdaBoost (AP=0.947), LR (AP=0.943), XGBoost (AP=0.940),DL-MLP (AP=0.938), SVM (AP=0.935), RF (AP=0.912), and DT (AP=0.760) (Figure 9).


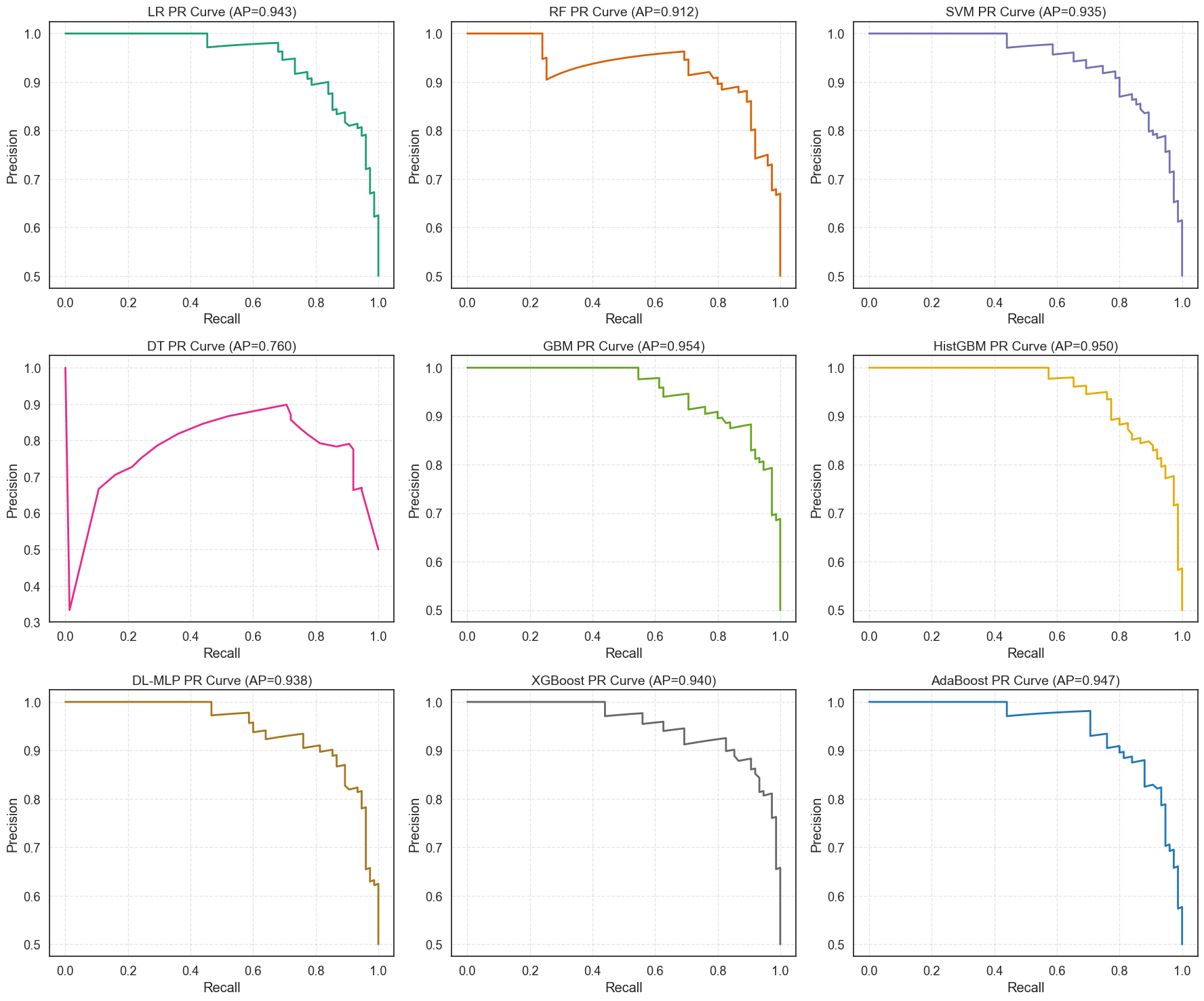


^Figure 12. Precision-recall curve is presented predict CSA practice for mushroom production based on nine ML algorithms with SMOTE, repeated stratified 10 fold CV with hyperparameter. The PR curve plots Recall (Sensitivity) on the X-axis and Precision (Positive Predictive Value) on the Y-axis. This curve evaluates how well the model balances minimizing false positives and maximizing true positives. A curve closer to the top-right corner indicates better performance in both precision and recall.^

In terms of predict CSA practice, the SVM algorithm showed high accuracy and g-mean score with relatively tight distributions, suggesting they are strong and stable performers than other algorithms (Figure 13).


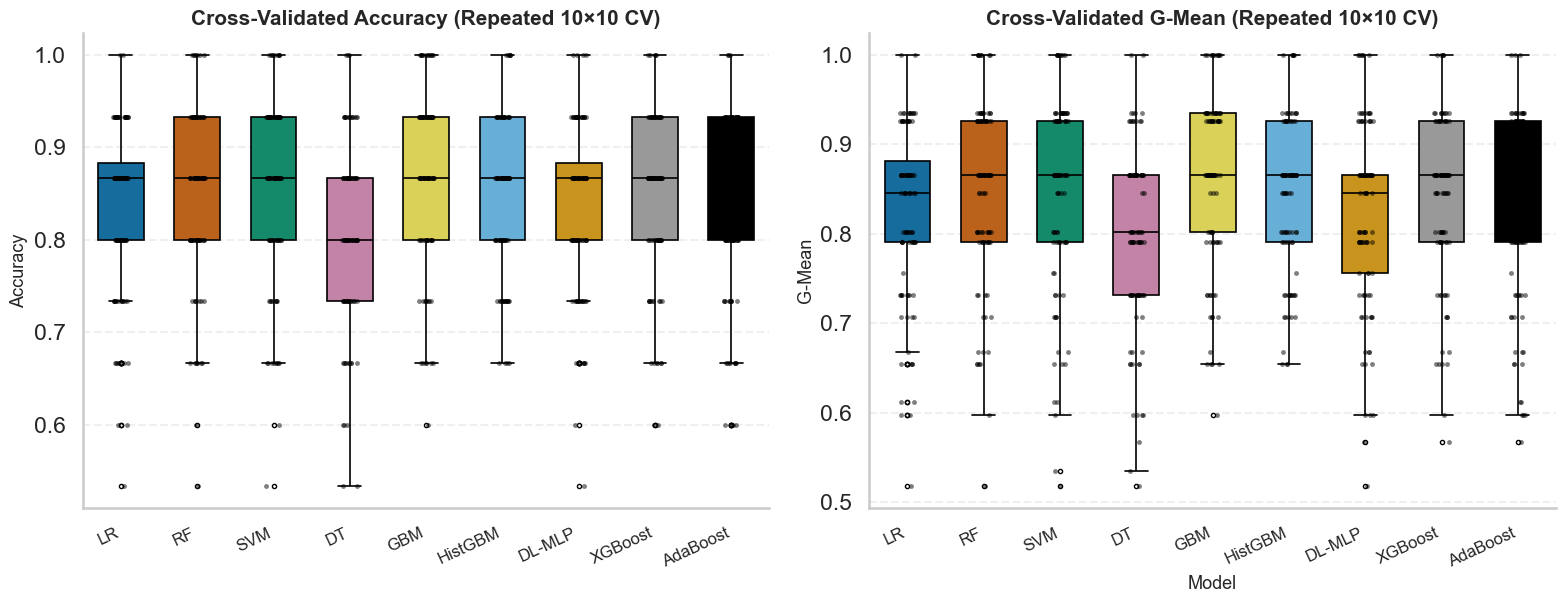


^Figure 13. shows box plots summarize the distribution of accuracy and g-mean score among our selected nine ML classifiers which used to predict CSA practice among mushroom farmer. The x-axis represents the algorithms, while the y-axis shows the accuracy (left panel) and g-mean score (right panel) values for each classifier. The central line each box indicates the median of the performance. The bars within the plot represent the interquartile range of the distribution. Additionally, a Kernel Density Estimation (KDE) plot is used to define the shape of the violin, illustrating the distribution of data points.^


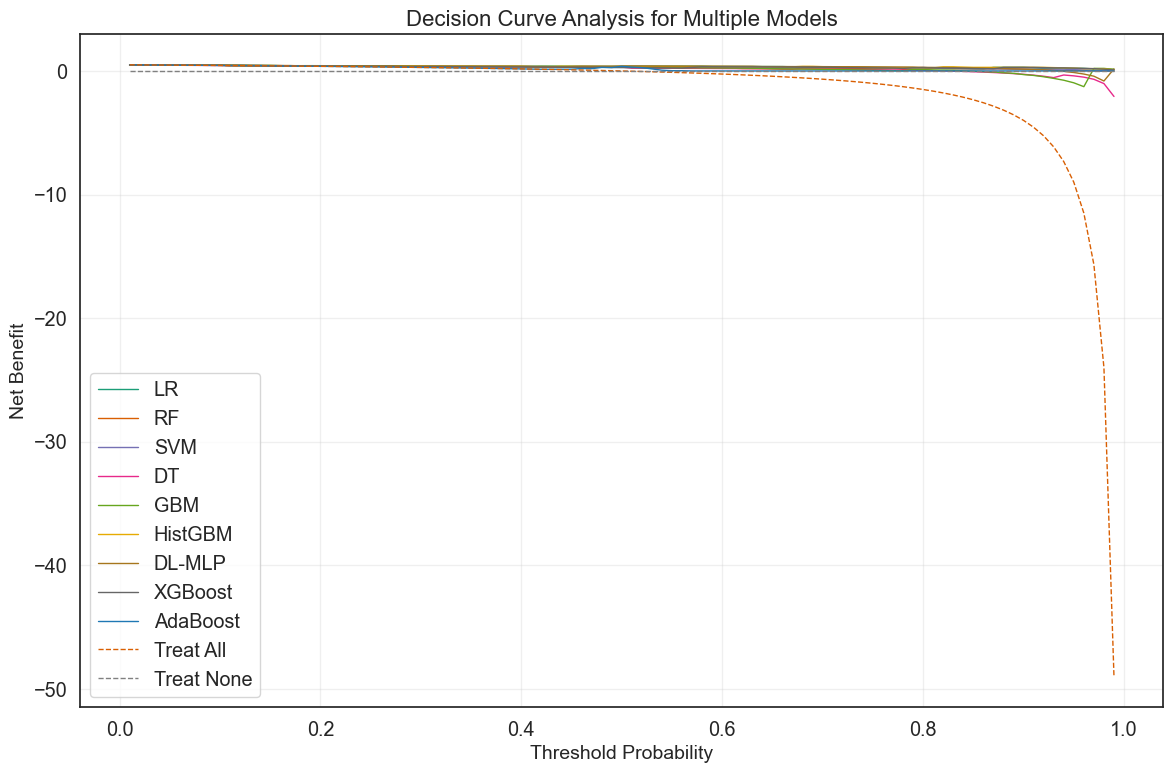


Figure 14. Decision Curve Analysis for Multiple Models

**Questionnaires (English)**

**Determinants of climate-smart agriculture (CSA) in mushroom farming: Dual application of Bayesian and machine learning approach**

**Consent: Yes/No**

Sample no: ______________ Date: ___________________

1. **Respondent’s General Information**:

Name: _________________________ Area: __________________

**Respondents socioeconomic Characteristics**:

Age(years): ___________ Gender: Male /Female

Education: No education/Primary/Secondary+

Main Occupation: __________________ other occupation (if any): _____________________

Farmer wealth Status……………….. Farming Experience (years):

Beneficiary to subsidiary program: Yes/ No

Household Size:

Health condition: Good/ Not good

1. **Characteristics of Mushroom farm**

| **Characteristics** | **Quantity** |
| --- | --- |
| Number of Mushroom Farms |  |
| Total Farm Size (Decimal) |  |
| Annual production of Mushroom farming (kg) |  |
| Annual cost of the mushroom farming |  |
| Annual profit of the mushroom farming |  |
| Experience in mushroom farming(Years) |  |

1. **Farmers knowledge on CSA technologies**

| Questions | Yes | No |
| --- | --- | --- |
| Do you know about CSA technologies |  |  |
| Do you think you are getting benefitted for using these technologies |  |  |
| Did you receive any training for Mushroom farming |  |  |

**4. Social outcomes**

| Frequency: | Farm-house distance (km): |
| --- | --- |
| Membership: Yes/ No | Food safety and security: Yes/ No |
| Mobile phone: Yes/ No |  |
| Internet: Yes/ No |  |

Do you know about relative humidity and ventilation for mushroom cultivation

| Do you know the appropriate temperature required for mushroom farming? Yes/ No | Do you know the importance of light in mushroom cultivation? Yes/ No |
| --- | --- |
| Do you know the importance of substrate (soil) quality in mushroom cultivation? Yes/ No | Do you know the optimal relative humidity range required for mushroom cultivation? (Yes/No) |
| Do you have access to climatic information (e.g., temperature, humidity) for mushroom cultivation? (Yes/No) | Personal and environmental health: Yes/ No |
| Soil quality: Yes/ No |  |
| Nutrient leaching: Yes/ No |  |

**5. Knowledge about Environmental outcomes**

**6. Institutional factors**

| Questions | **Yes** | **No** | Questions | **Yes** | **No** |
| --- | --- | --- | --- | --- | --- |
| Access to market |  |  | Local processing facilities |  |  |
| Contract farming |  |  | Transport availability |  |  |
| Training |  |  | Distance from nearest extension office (km): |  | |
| Local storage |  |  | Distance from district capital (km): |  | |
| Access to credit |  |  | Market distance (km) |  | |
|  |  |  | Extension visits number |  | |

**7. CSA Practice**

| **CSA practices** | **Put tick (√) mark on adopted practices** | |
| --- | --- | --- |
|  | **Yes** | **No** |
| Utilization of an autoclave for sterilizing mushroom substrates |  |  |
| Adoption of climate-friendly varieties (oyster mushrooms, and king oyster) |  |  |
| Use of high yielding mushroom varieties (Pleurotus*spp.* and Pleurotus eryngii) |  |  |
| Utilization of Internet of Things (IoT)-based monitoring and control systems |  |  |
| Use of organic fertilizer |  |  |
| Climate control via heating, ventilation, and air conditioning (HVAC) equipment |  |  |
| Adoption of solar energy (e.g., photovoltaic (PV) for mushroom cultivation to mitigation the climate warming |  |  |
| Use of electric sterilizers (steam) machines for bag filling |  |  |
| Humidity control via spraying water mist using spray |  |  |
| Utilization of integrated pest management (IPM) practices |  |  |
| Use of sprinklers for optimal humidity level |  |  |
| Use of media (sawdust and rice straw) as compost for mushroom production, |  |  |
| Use of spent mushroom substrate (SMS) method as an alternative of burying the media and polythene covers |  |  |

What are the major constraints?

Is there any CSAT that you want to adopt but cannot? If yes, then why?

**Questionnaires (English)**

**Determinants of climate-smart agriculture (CSA) in mushroom farming: Dual application of Bayesian and machine learning approach (মাশরুম চাষে জলবায়ু-বান্ধব কৃষি (CSA) নির্ধারক: বেসিয়ান ও মেশিন লার্নিং পদ্ধতির যৌথ প্রয়োগ)**

**সম্মতি**: হ্যাঁ / না

**নমুনা নম্বর: ______________ তারিখ: ___________________**

## উত্তরদাতার সাধারণ তথ্য:

নাম: _________________________

এলাকা: __________________

## উত্তরদাতার সামাজিক ও অর্থনৈতিক বৈশিষ্ট্য:

বয়স (বছর): ___________

লিঙ্গ: পুরুষ / মহিলা

শিক্ষা: অশিক্ষিত / প্রাথমিক / মাধ্যমিক বা তদূর্ধ্ব

প্রধান পেশা: __________________

অন্যান্য পেশা (যদি থাকে): _____________________

চাষীর সম্পদগত অবস্থা: ………………..

চাষের অভিজ্ঞতা (বছর): __________

সাবসিডি প্রোগ্রামের সুবিধাভোগী: হ্যাঁ / না

পরিবারের সদস্য সংখ্যা: __________

স্বাস্থ্য অবস্থা: ভালো / ভালো নয়

## মাশরুম খামারের বৈশিষ্ট্য:

| **বৈশিষ্ট্য** | **পরিমাণ** |
| --- | --- |
| মাশরুম খামারের সংখ্যা |  |
| মোট খামারের আয়তন (ডেসিমেল) |  |
| বার্ষিক উৎপাদন (কেজি) |  |
| বার্ষিক ব্যয় |  |
| বার্ষিক লাভ |  |
| মাশরুম চাষে অভিজ্ঞতা (বছর) |  |

## CSA প্রযুক্তি সম্পর্কে কৃষকের জ্ঞান:

| **প্রশ্ন** | **হ্যাঁ** | **না** |
| --- | --- | --- |
| আপনি কি CSA প্রযুক্তি সম্পর্কে জানেন? |  |  |
| আপনি কি মনে করেন এই প্রযুক্তিগুলো ব্যবহার করে আপনি উপকৃত হচ্ছেন? |  |  |
| আপনি কি মাশরুম চাষ বিষয়ে কোনো প্রশিক্ষণ পেয়েছেন? |  |  |

## সামাজিক ফলাফল:

| Frequency: | খামার থেকে বাড়ির দূরত্ব (কিমি): |
| --- | --- |
| সদস্যপদ (যেকোনো সংগঠনে): হ্যাঁ / না | খাদ্য নিরাপত্তা ও সুরক্ষা: হ্যাঁ / না |
| মোবাইল ফোন আছে কি না: হ্যাঁ / না |  |
| ইন্টারনেট আছে কি না: হ্যাঁ / না |  |

আপনি কি মাশরুম চাষের জন্য আপেক্ষিক আর্দ্রতা এবং বায়ুচলাচল সম্পর্কে জানেন?

1. **পরিবেশগত ফলাফল সম্পর্কে জ্ঞান:**

| মাশরুম চাষের জন্য উপযুক্ত তাপমাত্রার প্রয়োজন জানেন কি? হ্যাঁ/ না | মাশরুম চাষে আলোর গুরুত্ব জানেন কি? হ্যাঁ/ না |
| --- | --- |
| মাশরুম চাষে সাবস্ট্রেট (মাটি) গুণের গুরুত্ব জানেন কি? হ্যাঁ / না | আপনি কি মাশরুম চাষের জন্য প্রয়োজনীয় সর্বোত্তম আপেক্ষিক আর্দ্রতার পরিসীমা জানেন? (হ্যাঁ/না) |
| আপনার কি মাশরুম চাষের জন্য জলবায়ু সম্পর্কিত তথ্য (যেমন, তাপমাত্রা, আর্দ্রতা) অ্যাক্সেস আছে? (হ্যাঁ/না) | ব্যক্তিগত এবং পরিবেশগত স্বাস্থ্য: হ্যাঁ / না |
| মাটির গুণমান: হ্যাঁ / না |  |
| পুষ্টি ক্ষয় (nutrient leaching): হ্যাঁ / না |  |

## প্রাতিষ্ঠানিক উপাদান:

| প্রশ্ন | হ্যাঁ | না | প্রশ্ন | হ্যাঁ | না |
| --- | --- | --- | --- | --- | --- |
| বাজারে প্রবেশাধিকার |  |  | স্থানীয় প্রক্রিয়াকরণ সুবিধা |  |  |
| চুক্তিভিত্তিক চাষ: |  |  | পরিবহন ব্যবস্থা |  |  |
| প্রশিক্ষণ |  |  | নিকটবর্তী সম্প্রসারণ অফিস থেকে দূরত্ব (কিমি) |  |  |
| স্থানীয় সংরক্ষণ সুবিধা |  |  | জেলা সদর থেকে দূরত্ব (কিমি) |  |  |
| ঋণ সুবিধা |  |  | বাজারের দূরত্ব (কিমি) |  |  |
|  |  |  | সম্প্রসারণ পরিদর্শনের সংখ্যা: |  |  |

## CSA অনুশীলন:

| CSA পদ্ধতিগুলোতে গ্রহণযোগ্যতার চিহ্ন (√) দিন | | |
| --- | --- | --- |
| অটোক্লেভ ব্যবহার | হ্যাঁ | না |
| জলবায়ু-বান্ধব জাত(oyster mushrooms, and king oyster): |  |  |
| উচ্চ ফলনশীল জাত(Pleurotus*spp.* and Pleurotus eryngii): |  |  |
| IoT ভিত্তিক পর্যবেক্ষণ(monitoring and control systems) |  |  |
| জৈবসার ব্যবহার |  |  |
| হিটিং, বায়ুচলাচল এবং এয়ার কন্ডিশনার (এইচভিএসি) সরঞ্জামের মাধ্যমে জলবায়ু নিয়ন্ত্রণ |  |  |
| জলবায়ু উষ্ণায়ন প্রশমিত করার জন্য মাশরুম চাষের জন্য সৌর শক্তি গ্রহণ (উদাঃ, ফটোভোলটাইক (পিভি) |  |  |
| ব্যাগ ভর্তি জন্য বৈদ্যুতিক জীবাণুনাশক (বাষ্প) মেশিন ব্যবহার |  |  |
| স্প্রে ব্যবহার করে জলের কুয়াশা স্প্রে করার মাধ্যমে আর্দ্রতা নিয়ন্ত্রণ |  |  |
| বালাই ব্যবস্থাপনা (আইপিএম) অনুশীলনের ব্যবহার |  |  |
| মাশরুম উৎপাদনের জন্য কম্পোস্ট হিসাবে মিডিয়া (করাত এবং ধানের খড়) ব্যবহার |  |  |
| মিডিয়া এবং পলিথিন কভার কবর দেওয়ার বিকল্প হিসাবে ব্যয়িত মাশরুম সাবস্ট্রেট (এসএমএস) পদ্ধতির ব্যবহার |  |  |

প্রধান প্রতিবন্ধকতাগুলো কী কী?

এমন কোনো CSA প্রযুক্তি আছে যা আপনি গ্রহণ করতে চান কিন্তু পারেন না? যদি হ্যাঁ হয়, তাহলে কেন?
